# Sulfur-Polymer Nanoparticles: Preparation and Antibacterial Activity

**DOI:** 10.1021/acsami.3c03826

**Published:** 2023-04-19

**Authors:** Romy A. Dop, Daniel R. Neill, Tom Hasell

**Affiliations:** † Department of Chemistry, University of Liverpool, Liverpool L69 7ZD, United Kingdom; ‡ Department of Clinical Infection, Microbiology and Immunology, Institute of Infection, Veterinary and Ecological Sciences, University of Liverpool, Liverpool L69 7ZD, United Kingdom; § College of Chemistry and Chemical Engineering, Gansu International Scientific and Technological Cooperation Base of Water-Retention Chemical Functional Materials, Northwest Normal University, Lanzhou 730070, P. R. China

**Keywords:** inverse vulcanization, sulfur, polysulfides, antibacterial, biofilm inhibition, nanoparticles

## Abstract

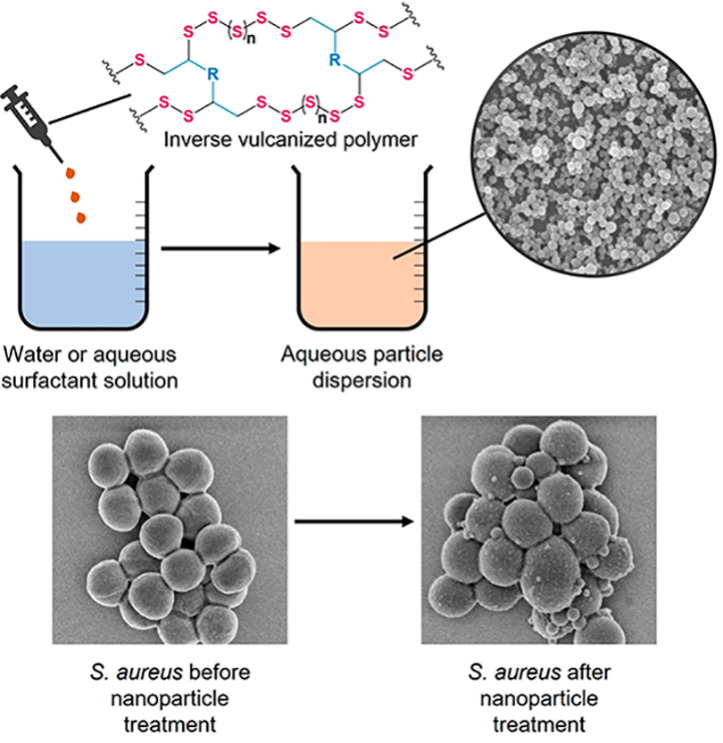

High sulfur content polymers prepared by inverse vulcanization have many reported potential applications, including as novel antimicrobial materials. High sulfur content polymers usually have limited water-solubility and dispersibility due to their hydrophobic nature, which could limit the development of their applications. Herein, we report the formulation of high sulfur content polymeric nanoparticles by a nanoprecipitation and emulsion-based method. High sulfur content polymeric nanoparticles were found to have an inhibitory effect against important bacterial pathogens, including Gram-positive methicillin-resistant *Staphylococcus aureus* and Gram-negative *Pseudomonas aeruginosa*. Salt-stable particles were formulated with the addition of a surfactant, which did not inhibit the antibacterial activity of the polymeric particles. Furthermore, the polymeric nanoparticles were found to inhibit *S. aureus* biofilm formation and exhibited low cytotoxicity against mammalian liver cells. Interaction of the polymeric particles with cellular thiols could be a potential mechanism of action against bacterial cells, as demonstrated by reaction with cysteine as a model thiol. The findings presented demonstrate methods of preparing aqueous dispersions of high sulfur content polymeric nanoparticles that could have useful biological applications.

## Introduction

**Figure 1:**
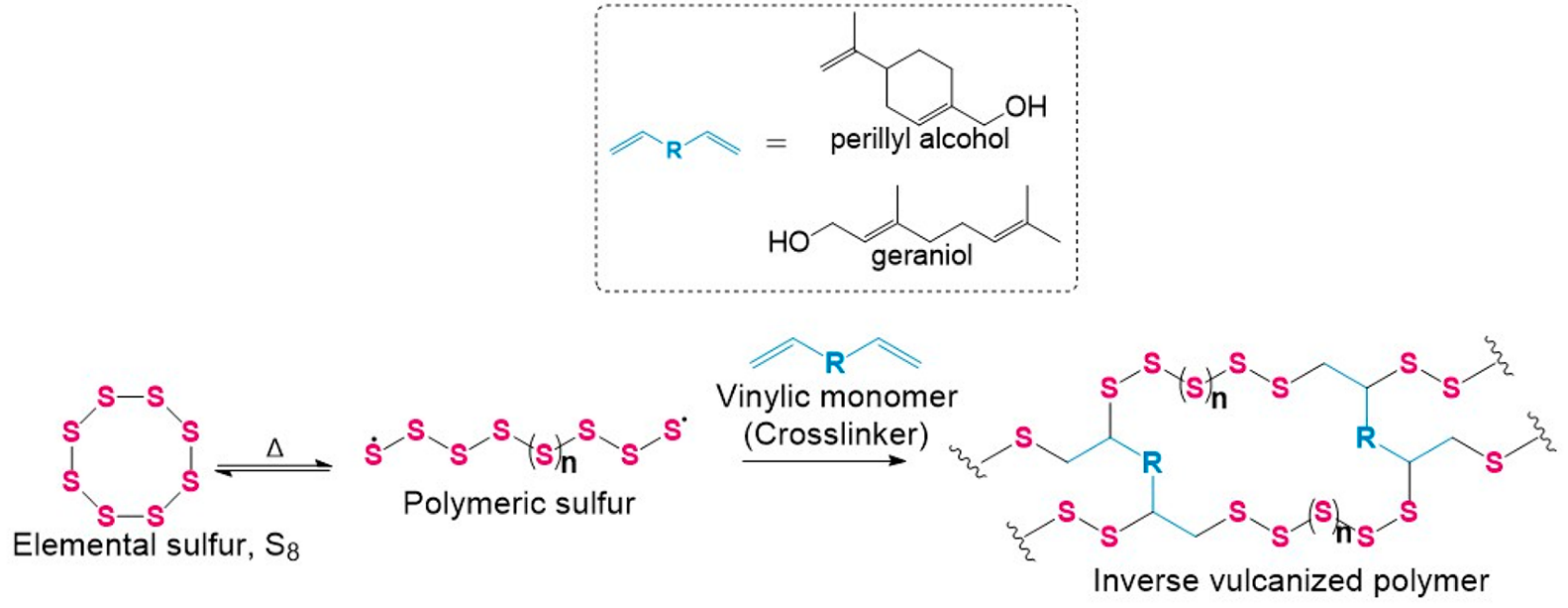
General scheme for the synthesis of copolymers from elemental sulfur and simple organic cross-linkers by inverse vulcanization, with the structures of the comonomers perillyl alcohol and geraniol shown.

High sulfur content polymers (denoted S-comonomer) with >50 wt % sulfur can be prepared by inverse vulcanization (Figure 1), a term coined by Pyun and colleagues in 2013.^4^ Inverse vulcanization consists of polymerizing elemental sulfur with a vinylic comonomer. The method is a bulk polymerization that does not require a solvent. Since 2013, a large library of compatible monomers has been reported, including dicyclopentadiene, 1,3-diisopropenylbenzene, and divinylbenzene.^4−3^ Furthermore, the polymers can be made from naturally derived products, using vegetable oils or terpenes as the comonomers.^5−7^ High sulfur content polymers have been reported to have potential applications in heavy metal remediation,^7^ oil removal,^8^ novel optical devices,^9^ and as smart fertilizers.^10^ In addition to this, there is interest in high sulfur content polymers as potential antimicrobial materials. In 2017, Deng et al. showed the inhibition of *Escherichia coli* by thin layers of poly(sulfur-*co*-1,3-diisopropenyl benzene) (S-DIB) spin-coated onto silicon substrates.^1^ Furthermore, Cubero-Cardoso and colleagues synthesized high sulfur content polymers using vegetable oils and additional additives for the preparation of antimicrobial and antioxidant materials.^11^ Recently, Upton et al. reported the fabrication of water-repellent coatings using high sulfur content polymers and silica that were found to inhibit *Staphylococcus aureus*.^12^

High sulfur content polymers are often hydrophobic and have limited water solubility. Preparing high sulfur content polymeric nanoparticles could provide a method to increase the water dispersibility of the polymers and to increase the surface area of the material, which could be beneficial for applications in oil adsorption, heavy metal uptake, and novel antimicrobials. Lim and co-workers reported the *in situ* synthesis of water dispersible high sulfur content nanoparticles by the interfacial polymerization of sodium polysulfide and 1,2,3-trichloropropane in water. Dynamic light scattering (DLS) measurements showed nanoparticles with an average hydrodynamic diameter of 172.8 ± 33.1 nm.^13^ More recently, Zhang et al. demonstrated that dispersions of high sulfur content polymer nanoparticles can be formed in ethanol using a nanoprecipitation, also known as a solvent/antisolvent precipitation, method.^14^ The obtained dispersions were found to be able to bind selectively to mercury in a mixed ion solution, showing potential for the high sulfur content polymer nanoparticles to be used for environmental remediation purposes.^14^ Antibacterial polymeric nanoparticles have found applications as wound dressings, antimicrobial surface coatings, and as drug delivery systems. High sulfur content polymers have been found to have antibacterial activity;^15^ however, the bulk materials often have low water dispersibility.^13^ To our knowledge, the antibacterial activity of inverse vulcanized polymeric nanoparticles has not been investigated. Formulating polymeric nanoparticles of high sulfur content polymers could expand the potential applications for high sulfur content polymers with regards to their antibacterial activity.

Herein, we report the preparation of water-dispersible high sulfur content polymer nanoparticles prepared by emulsion-templated and nanoprecipitation methods. The antibacterial activity of the polymeric nanoparticles was assessed against Gram-positive methicillin-resistant *S. aureus* and Gram-negative *Pseudomonas aeruginosa*. Biofilm inhibition on the surface of containers was also investigated upon the addition of high sulfur content nanoparticles. Cytotoxicity of the polymeric nanoparticles was evaluated with mammalian liver cells (HepG2 cell line). Current studies on the antibacterial activity of high sulfur content polymers have not identified the potential mechanisms of action of the polymers. Herein, we show that interaction of the polymeric particles with cellular thiols is a potential mechanism of action, as demonstrated by using cysteine as a model thiol.

## Experimental Details

### Materials and Equipment

#### Materials

Ground sulfur sublimed powder reagent grade ≥99.5% was obtained from Brenntag U.K. and Ireland. (*S*)-(−)-Perillyl alcohol food grade ≥95%, geraniol food grade ≥97%, Luria–Bertani broth (Miller), LB agar and phosphate buffered saline (PBS), crystal violet, cell proliferation kit I (MTT), and lead(II) acetate test strips were purchased from Sigma-Aldrich. Eagle’s minimum essential medium (EMEM) was purchased from ATCC. Methicillin-resistant *S. aureus* strain USA300 and *P. aeruginosa* strains PAO1 and B9 were cultured from frozen stocks stored at the University of Liverpool.

#### Differential Scanning Calorimetry (DSC)

DSC was performed using a TA Instruments Q200 DSC, programmed using a heat/cool/heat method for three cycles by heating to 150 °C, cooling to −80 °C, and reramping to 150 °C. The heating/cooling rate was set to 10 °C/min. The second heating curve was analyzed and used to determine the glass transition temperature.

Fourier transformed infrared spectroscopy (FT-IR) data was obtained on a Bruker TENSOR 27 FT-IR, between 400 and 4000 cm^–1^ using an attenuated total reflectance accessory.

Nuclear magnetic resonance (NMR) samples were analyzed using a Bruker Advance DRX (400 MHz) spectrometer using deuterated chloroform as the solvent; all experiments were carried out at room temperature.

Dynamic light scattering (DLS) measurements were obtained at 25 °C on a Malvern Instruments Ltd. Zetasizer Nano Series Nano-ZS spectrometer using the automatic attenuator and measurement position settings. The z-average diameter was measured using 1 cm path length disposable cuvettes. Nanoparticles were dispersed at a range of concentrations to determine a size independent of the concentration.

Scanning electron microscopy (SEM) was performed using a Hitachi S-4800 cold-field emission scanning electron microscope. The nanoparticle dispersions were dropped onto silicon wafer chips, which were subsequently mounted onto SEM stubs using conductive silver paint. For imaging cells after treatment with nanoparticles, diluted *S. aureus* cultures supplemented with nanoparticles, or water as a control, were incubated for 5 h. The cultures were pelleted by centrifugation and washed with PBS. The pellets were incubated overnight in glutardialdehyde and then dispersed and spun down in a series of ethanol dilutions (50, 70, 90, 95, and 100% v/v). The pellets were redispersed in ethanol, and the resulting dispersions were dropped onto silicon wafers. Prior to imaging, samples were coated with gold using a current of 120 mA for 15 s to give approximately 15 nm gold coatings using a Quorum S1505 ES sputter coater.

Absorbance measurements for the MTT assay were obtained using a BMG Labtech FLUOstar Omega microplate reader using 96-well plates.

### Synthesis of High Sulfur Content Polymers

Polymerization was carried out in 40 mL glass vials placed in aluminum heating blocks. Sulfur/cross-linker weight ratios of 50 and 70 wt % sulfur were used, with the total reaction scale maintained at 10 g. All reactions were begun by allowing the sulfur to melt at 135 °C before adding the organic cross-linker under stirring. The reaction temperature was increased to 175 °C. Molten prepolymer was poured into silicone molds followed by curing overnight in an oven at 140 °C. The mixture was transferred from the stirred vial to the mold when the reaction mixture had become homogeneous and viscous (an aliquot of the reaction mixture, when removed on a spatula and allowed to cool to room temperature, would no longer visibly separate to clear organic monomer and precipitated yellow sulfur powder). The cured polymers were allowed to cool and were ground into powders using a pestle and mortar.

### Preparation of Polymeric Nanoparticles

#### Emulsion-Solvent Evaporation Method

Nine milliliters of aqueous surfactant solution (varying concentrations) was added to a 14 mL glass vial. One milliliter of S50-Ger in chloroform (5 mg/mL, 50 wt % S) was added to the vial and immediately sonicated for 40 s. The vial was equipped with a 15 mm × 6 mm magnetic stirrer bar, and the resulting cream-colored emulsion was allowed to stir at 600 rpm and at room temperature overnight or until the chloroform had evaporated.

#### Nanoprecipitation Method

Nine milliliters of aqueous surfactant solution (10 mg/mL) or distilled water was added to a 14 mL glass vial and allowed to stir at room temperature at 600 rpm. One milliliter of S-Ger or S-PA in tetrahydrofuran (THF) (varying concentrations, 50 and 70 wt % S) was added to the aqueous solution dropwise with continued stirring at 600 rpm. Once 1 mL of the polymer dissolved in THF was added to the vial, the solution was continued to stir at room temperature at 600 rpm overnight or until THF had evaporated fully.

### Bacteria Preparation, Storage, and Enumeration

Glycerol stocks of *S. aureus* strain USA300 and *P. aeruginosa* strain PAO1 were stored at −80 °C for long-term storage. For experimental use, frozen glycerol stocks of *S. aureus* and *P. aeruginosa* were defrosted and spread onto LB agar plates, which were then incubated overnight at 37 °C. Bacterial cultures were prepared by swabbing one colony into 10 mL of LB broth, followed by overnight incubation at 37 °C. Colony forming units (CFUs) were enumerated by serially diluting the cultures in PBS onto LB agar, using the Miles and Misra method. CFU/cm^2^ and CFU/mL were calculated using the following equation:
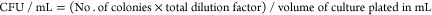


### Viable Bacterial Cell Enumeration Assay

*S. aureus* USA300 and *P. aeruginosa* PAO1 were used to evaluate the antibacterial efficiency of the high sulfur content polymeric nanoparticles. Blank samples were prepared by dropping 1 mL of THF into 9 mL of water followed by overnight stirring (600 rpm) at room temperature to allow for the THF to evaporate. Overnight cultured bacteria prepared in LB broth were diluted to 10^5^ CFU/mL (OD600 = 0.001) in LB or M9 media. Nine hundred microliters of diluted bacterial solution was added to 2 mL vials along with 100 μL of nanoparticles dispersed in water (final concentration of nanoparticles: 14, 27, 55, 220, and 440 μg/mL) or blank. The samples were incubated for 5 h at 37 °C on a roller. Viable cells were enumerated after serial dilution of the solution in PBS onto LB agar, using the Miles and Misra method at 0, 10, 30, 60, 90, 120, and 300 min.

### Determination of the Minimum Inhibitory Concentration

Minimum inhibitory concentrations of sulfur nanoparticles were assessed according to the European Committee on Antimicrobial Susceptibility Testing (EUCAST) guidelines,^16^ for an incubation period of 24 h against *S. aureus* strain USA300 and *P. aeruginosa* strain PAO1, in LB medium. An initial OD600 of 0.1 (∼5 × 10^5^ CFU/mL) was used for the cell cultures prior to incubation. Nanoparticles were tested at 2-fold dilutions spanning a concentration range of 0.5–512 μg/mL. The OD600 was measured using a FLUOstar Omega microplate reader. The same method was used to investigate the antibacterial activity of the nanoparticles, combined with tobramycin, against the extensively drug resistant *P. aeruginosa* strain B9, isolated from an acute respiratory infection in Thailand.^17^

### Disc Diffusion Assay

*S. aureus* USA300 and *P. aeruginosa* PAO1 were used to evaluate the antibacterial efficiency of the high sulfur content polymeric nanoparticles using the disc diffusion assay. Overnight cultured bacteria prepared in LB broth was streaked onto LB agar plates. Blank antimicrobial susceptibility testing discs were soaked with 50 μL of nanoparticles. A control sample was prepared by soaking the empty discs with 50 μL of water. The discs were placed on top of the lawn of bacteria, and the plates were incubated for 24 h at 37 °C.

### Biofilm Staining Assay

*S. aureus* USA300 and *P. aeruginosa* PAO1 were used to evaluate biofilm formation in the presence of high sulfur content polymeric nanoparticles. Blank solutions were prepared by dropping 1 mL of THF into 9 mL of water followed by overnight stirring (600 rpm) at room temperature to allow for the THF to evaporate. Overnight cultured bacteria prepared in LB broth was diluted to 10^5^ CFU/mL (OD600 = 0.001). Nine hundred microliters of diluted culture and 100 μL of nanoparticles dispersed in water (final concentrations of 220 and 440 μg/mL) or blank solution were added to separate wells of a 24-well plate. The well plate was incubated statically at 37 °C for 24 and 48 h. After incubation, the solutions from the well plate were discarded and it was rinsed with 1 mL of PBS, which was then discarded and the plate was allowed to dry. The dried wells were stained with 1 mL 0.25% crystal violet for 30 min. The dye was discarded, and the well plate was thoroughly rinsed with water and allowed to dry. One milliliter of ethanol was added to each well in order to solubilize any remaining dye. The absorbance at 600 nm was measured using ethanol as a blank.

### Cell Culture

HepG2 cells were maintained in Eagle’s minimum essential medium (EMEM) cell culture medium (ATCC) supplemented with 10% fetal bovine serum (FBS). Cells were maintained in a 5% CO_2_ incubator at 37 °C.

### Cell Viability Assay

Cell viability was evaluated using the MTT assay. HepG2 cells were seeded at a concentration of 5 × 10^4^ cells/well in 100 μL culture medium and incubated (5% CO_2_, 37 °C) for 48 h or until approximately 80% confluent. Culture media was removed and replaced with 90 μL of culture medium and 10 μL of nanoparticles (final concentration of nanoparticles: 14, 27, 55, 220, and 440 μg/mL) or blank solution. The cells in the presence of nanoparticles/blank were incubated for 24 h (5% CO_2_, 37 °C). After 24 h of incubation, the media containing nanoparticles/blank was removed and replaced with 100 μL of culture medium and 10 μL of the MTT labeling reagent (final concentration of 0.5 mg/mL). The microplate was incubated for a further 4 h (5% CO_2_, 37 °C). One hundred microliters of the solubilizing buffer was added to each well, and the microplate was allowed to stand overnight in the incubator. The solubilization of the purple formazan crystals was measured at an absorbance wavelength of 595 nm.

### Statistical Analysis

Statistical analysis was conducted using one-way analysis of variance (ANOVA) followed by Tukeys post hoc test. Differences were deemed as statistically significant if a value of *p* < 0.05 was obtained.

### Cysteine-Mediated H_2_S Release

l-Cysteine (5 mg/mL) in PBS was added to an equal volume of S50-PA nanoparticles (5 mg/mL) in water in a 14 mL glass vial. Control solutions were prepared by adding l-cysteine to water and adding water to S50-PA nanoparticles. Stirrer beads were added to the vials, and approximately 1 cm of lead(II) acetate test paper was attached to the inside of the lid of the vial. The vials were allowed to stir over a period of 5 h at room temperature.

## Results and Discussion

### Polymer Synthesis

**Figure 2:**
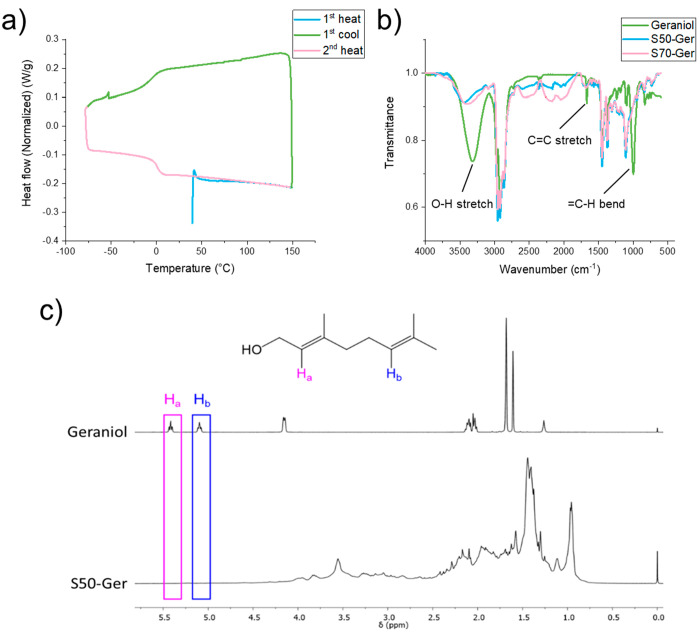
(a) DSC traces for S50-Ger showing the first heating cycle to 150 °C (blue), cooling to −80 °C (green), and the second heating cycle to 150 °C (pink). (b) FTIR spectra of geraniol (green), S50-Ger (blue), and S70-Ger (pink). (c) ^1^H NMR spectra of geraniol and S50-Ger.

Two polymers were chosen to prepare high sulfur content polymeric nanoparticles; the product of the copolymerization of sulfur and perillyl alcohol (S-PA) and the product of the copolymerization of sulfur and geraniol (S-Ger) (Figure 1). Perillyl alcohol and geraniol are both naturally derived terpenes that are found in the essential oils of lavender and geranium, among others.^18,19^ Copolymerization of elemental sulfur with perillyl alcohol or geraniol was carried out by adding the terpene monomers to molten sulfur at 135 °C and further heating at 175 °C until the mixture became homogeneous and viscous. The reaction mixture was cured overnight at 140 °C. Following curing, the polymers were ground into powders using a pestle and mortar. ^1^H nuclear magnetic resonance (NMR) and Fourier-transform infrared (FTIR) spectroscopy were used to evaluate the reaction by probing the signals corresponding to alkene units of the monomer. The *T*_g_ of the resultant materials was determined by differential scanning calorimetry (DSC). DSC was also used to determine the presence of any unreacted crystalline sulfur within the materials. The reaction of sulfur with geraniol yielded black pliable materials that become brittle after refrigeration (97% yield). DSC analysis of S50-Ger showed a *T*_g_ of 5.8 °C, indicating the presence of a polymeric material (Figure 2a). A loss of alkene signals in both the FTIR (Figure 2b) and ^1^H NMR spectra (Figure 2c) of S50-Ger suggest successful reaction between sulfur and geraniol at the carbon atoms of the alkene. S50-Ger (Table S1) was found to be soluble in chloroform, THF, and toluene and was found to have some solubility in acetone (Figure S1). The presence of a soluble fraction suggests that S50-Ger is not a fully cross-linked polymer. Maladeniya et al. found that cyclic microstructures can be found during the polymerization of sulfur with geraniol, which would lower the cross-link density of the polymer and thus allow for solubility in organic solvents.^20^ S50-PA has been previously synthesized and characterized, where it was found to exhibit an inhibitory effect against methicillin-resistant *S. aureus* and *P. aeruginosa*.^15^ The *T*_g_ of S50-PA was found to be 34 °C and similarly to S50-Ger; it was found to be soluble in both chloroform and THF.^15^

### Formulation of High Sulfur Content Polymer Nanoparticles

The solubility of S-PA and S-Ger in chloroform and THF is ideal for the formulation of nanoparticles by techniques that require preformed polymers. The solubility of both polymers in these solvents also allowed for two methods for nanoparticle formulation to be investigated, namely, the nanoprecipitation method and the emulsion-solvent evaporation method. S50-Ger was used to formulate nanoparticles by both methods. Water-based coatings are more desirable than solvent-based coatings due to the potential harmful effects of toxic solvents upon the environment; therefore, water was chosen as the antisolvent for both methods. The dispersions were analyzed by dynamic light scattering (DLS), where the main parameters measured were the z-average diameter and the polydispersity index (PDI).

An emulsion-solvent evaporation method for the preparation of polymeric nanoparticles was investigated for S-Ger. For this method, an oil-in-water (chloroform/water) emulsion was formed whereby the polymer was dissolved in the oil phase and a suitable surfactant was dissolved in the water phase. Various emulsifiers were trialled, including Tween 80, poly(vinyl alcohol) (PVA), Brij S20, and sodium dodecyl sulfate (SDS) (Figure S2). Tween 80 and Brij S20 are both nonionic surfactants with a hydrophile–lipophile balance of 15; PVA is a nonionic polymer that is often used as an emulsifier whereas SDS in an anionic surfactant.

The trial formulation for the preparation of S50-Ger dispersions consisted of a concentration of 5 mg/mL of polymer in chloroform and 10 mg/mL of surfactant in water, with an oil to water ratio of 1:9. A formulation without the addition of a surfactant was also trialled; upon ultrasonication of this mixture, a cream colored emulsion was formed which phase-separated immediately as expected. For this reason, a formulation without surfactant was deemed unsuitable for the preparation of polymeric nanoparticles. Stable emulsions were formed with all surfactants trialled, which were then allowed to stir at room temperature to allow for the evaporation of chloroform. The resulting dispersions were cream colored with no visible aggregates (Figure S3). DLS analysis of the dispersions formed with both Brij S20 and SDS were multimodal with poor quality correlograms suggesting particle aggregation (Figures S4 and S5); thus, Brij S20 and SDS were deemed unsuitable as surfactants for the preparation of S50-Ger nanoparticles. DLS of the nanodispersions of S50-Ger prepared with PVA as an emulsifier gave monomodal distributions at various dilutions with a z-average diameter of 367.7 nm and a PDI of 0.173 (Figure S6). The dispersions were found to be stable for up to 14 days (Figure S7).

Analysis of the dispersions formulated using Tween 80 gave smaller particle sizes compared to formulations with PVA (Figure S8). The difference in the nanoparticle size when formulated with PVA and Tween80 could be due to the ability of the surfactants to stabilize the emulsion in the first step before solvent evaporation. Optical microscopy was used to look at the emulsions formed in the first step of the emulsion-solvent evaporation method. Smaller oil droplets were formed when employing Tween80 as a surfactant compared to PVA (Figure S9).

The formulations contain a large excess of surfactant at a concentration of 10 mg/mL; therefore, reducing the concentration of surfactant in the aqueous phase was investigated. The critical micelle concentration (CMC) of Tween 80 is 0.015 mg/mL; therefore, concentrations that were closer to the CMC were chosen to be investigated. Dispersions of S50-Ger with Tween 80 at concentrations of 0.05 and 0.1 mg/mL were trialled. The presence of large aggregates was visible in both dispersions formed, and DLS analysis confirmed that both concentrations were unsuitable for S50-Ger nanoparticle preparation (Figures S10 and S11).

**Figure 3:**
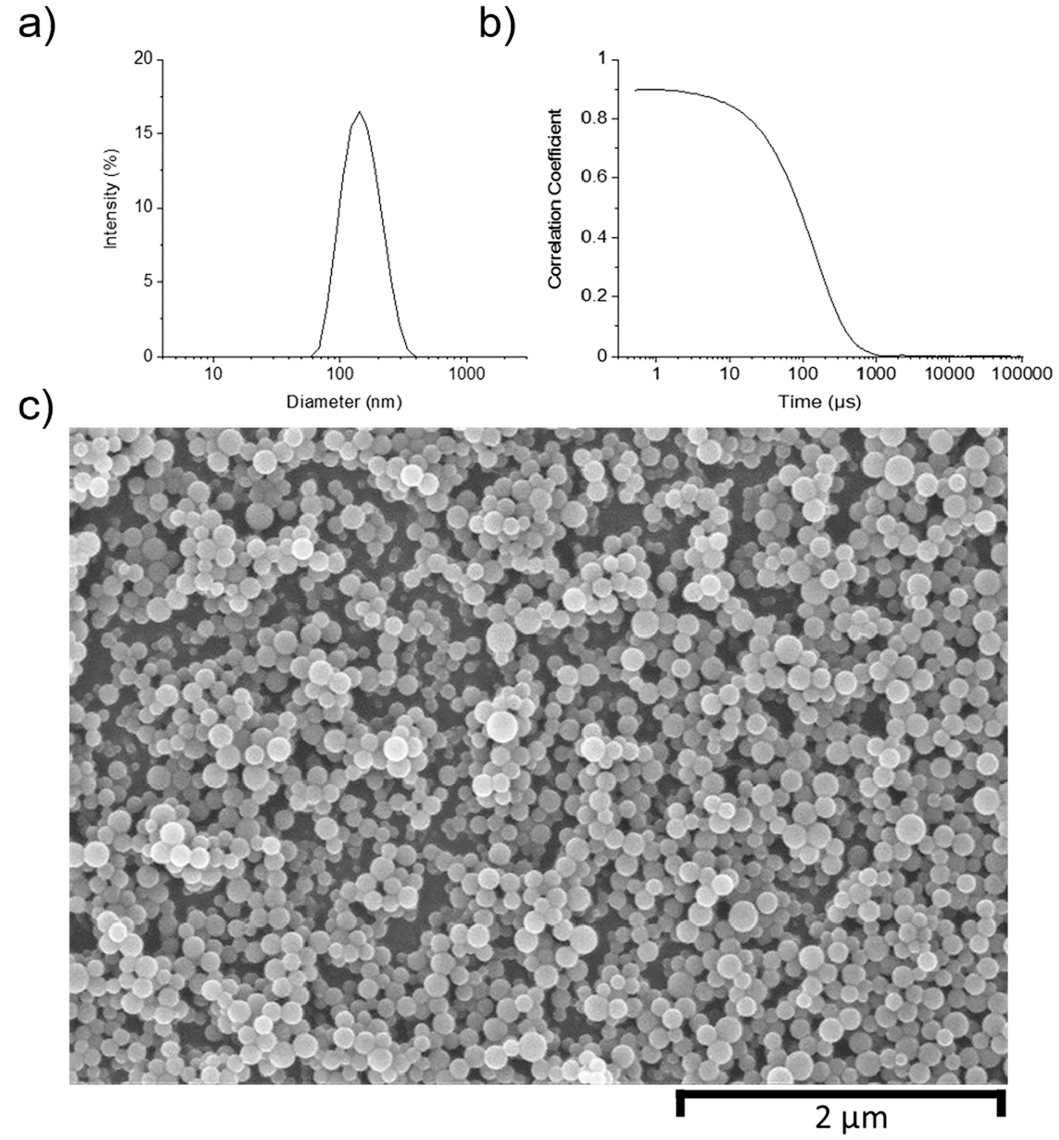
(a) Size distribution by intensity and (b) correlogram traces obtained for dispersions of S50-PA formed by a nanoprecipitation method without a surfactant. (c) SEM image of S50-PA dispersion.

A nanoprecipitation method was also investigated for inverse vulcanized polymer nanoparticles; THF was chosen as the solvent for the polymer and water as the antisolvent. The nanoprecipitation method does not require the use of a surfactant; therefore, a surfactant-free dispersion was formulated to establish whether S50-Ger polymer nanoparticles could be formed. The trial formulation consisted of 5 mg/mL polymer dissolved in THF, with a THF to water ratio of 1:9. After evaporation of THF, a cream colored cloudy solution remained with no visible aggregates. The size distribution by intensity obtained by DLS indicates the successful synthesis of polymeric nanoparticles with monomodal distributions (Figure S12). The particles obtained have a z-average diameter of 138 nm and a relatively low PDI of 0.165. The effect of the concentration of polymer dissolved in THF was studied by employing concentrations of 5, 10, and 20 mg/mL (Figure S13). The smallest particles were obtained with the lowest concentrations of polymer, and increasing the concentration results in an increase in both the nanoparticle z-average diameter and the PDI. In order to establish if the nanoprecipitation method can be used to formulate dispersions of other inverse vulcanized polymers, the method was repeated for S50-PA. DLS analysis of the dispersions formed in the absence of a surfactant using S50-PA at a concentration of 5 mg/mL in THF gave particles with monomodal distribution, a z-average diameter of 142.5 nm, and a PDI of 0.148 (Figure 3a,b). SEM images of the nanoparticles (Figure 3c) show that the particles are spherical and uniformly sized, consistent with the DLS data. The obtained sizes of the particles are very similar to those obtained with S50-Ger formulated at the same concentration of polymer in THF. This suggests that the nanoprecipitation method could be suitable to formulate nanoparticles of a range of inverse vulcanized polymers provided the polymer has good solubility in a water miscible solvent such as THF. It also shows that surfactant-free formulations are possible, which are particularly desirable for avoidance of additional purification steps.

Although the nanoprecipitation method does not require the use of a surfactant, it has been reported that employing a surfactant can often result in enhanced stability of the dispersion and smaller nanoparticles.^21^ Dispersions of S50-Ger by the nanoprecipitation methods were repeated using aqueous solutions of Tween 80 at a concentration of 10 mg/mL as the antisolvent. The z-average diameter of the particles formed using Tween 80 was similar to particles formed in the absence of a surfactant when employing a polymer concentration of 5 mg/mL (Figure S14). Similarly to the surfactant-free dispersions, the z-average diameter and PDI tended to increase with increasing polymer concentration in THF. However, at higher concentrations, the dispersions formed using Tween 80 have z-average diameters that are lower than those of the surfactant-free dispersions at the same polymer concentration. It is thought that the addition of a surfactant increases the viscosity of the dispersant and can cover the polymeric nanoparticles providing steric stabilization to prevent coalescence and aggregation of particles and resulting in smaller average sizes.^22^

Nanoparticles are often instable in the presence of salts, which can screen the charges of the nanoparticles, leading to particle–particle interactions and thus aggregation.^23^ To probe the effect of salt concentration on the stability of S50-PA nanoparticles, the average hydrodynamic diameter and ζ potential of the particles was measured in various NaCl solutions. NaCl solutions at concentrations of 1, 5, 25, 50, and 100 g/L were prepared, within which the nanoparticles were dispersed. The average hydrodynamic diameter and ζ potential of the nanoparticles were measured at 0, 1, and 7 days (Figure S15). Particles in the presence of >5 g/L NaCl were found to have immediately aggregated and were deemed unsuitable for DLS measurements, whereas particles in the presence of 5 g/L NaCl showed signs of aggregation due to the presence of a peak at higher particle diameters. S50-PA particles in the presence of 1 g/L NaCl were found to be stable during the 7 day period.

S50-PA polymeric nanoparticles formulated with Tween80 were prepared to investigate if the inclusion of a nonionic surfactant could provide stability to the nanoparticles against salts. S50-PA was dissolved to 5 mg/mL in THF and injected into aqueous Tween 80 at a concentration of 10 mg/mL (THF/water ratio of 1:9) under stirring. The solution was allowed to stir overnight at room temperature to allow THF to evaporate. The resulting dispersion was analyzed by DLS, which gave z-average diameters of (110 ± 2) nm, similar to S50-PA nanoparticles formulated without Tween80. NaCl solutions at concentrations of 1, 5, 25, 50, and 100 g/L were prepared, within which the nanoparticles in Tween80 were dispersed. S50-PA particles in Tween80 were found to be stable to all NaCl solutions after 7 days (Figure S16). The ζ potential of S50-PA nanoparticles with and without Tween80 in NaCl solutions were analyzed. In the absence of NaCl, S50-PA nanoparticles formulated without Tween80 have ζ potentials of (−56 ± 1) mV (Figure S17). It is possible that S50-PA nanoparticles are charge-stabilized due to having a large negative ζ potential.^24^ The addition of Tween80 in the formulation of the nanoparticles results in a less negative ζ potential of (−31 ± 2) mV. Tween80 is a nonionic surfactant and, thus, if adsorbed onto the surface of S50-PA nanoparticles is expected to reduce the magnitude of the ζ potential of the nanoparticles.^25^ When diluted in salt solutions containing NaCl at 1 and 5 g/L, the ζ potentials of the nanoparticles with and without Tween80 are reduced further in magnitude. The higher concentration of NaCl results in higher ionic strength of the dispersive medium, which can screen the charges of the nanoparticles and thus reduce the electrostatic repulsion forces between particles.^26^

### Antibacterial Activity

**Figure 4:**
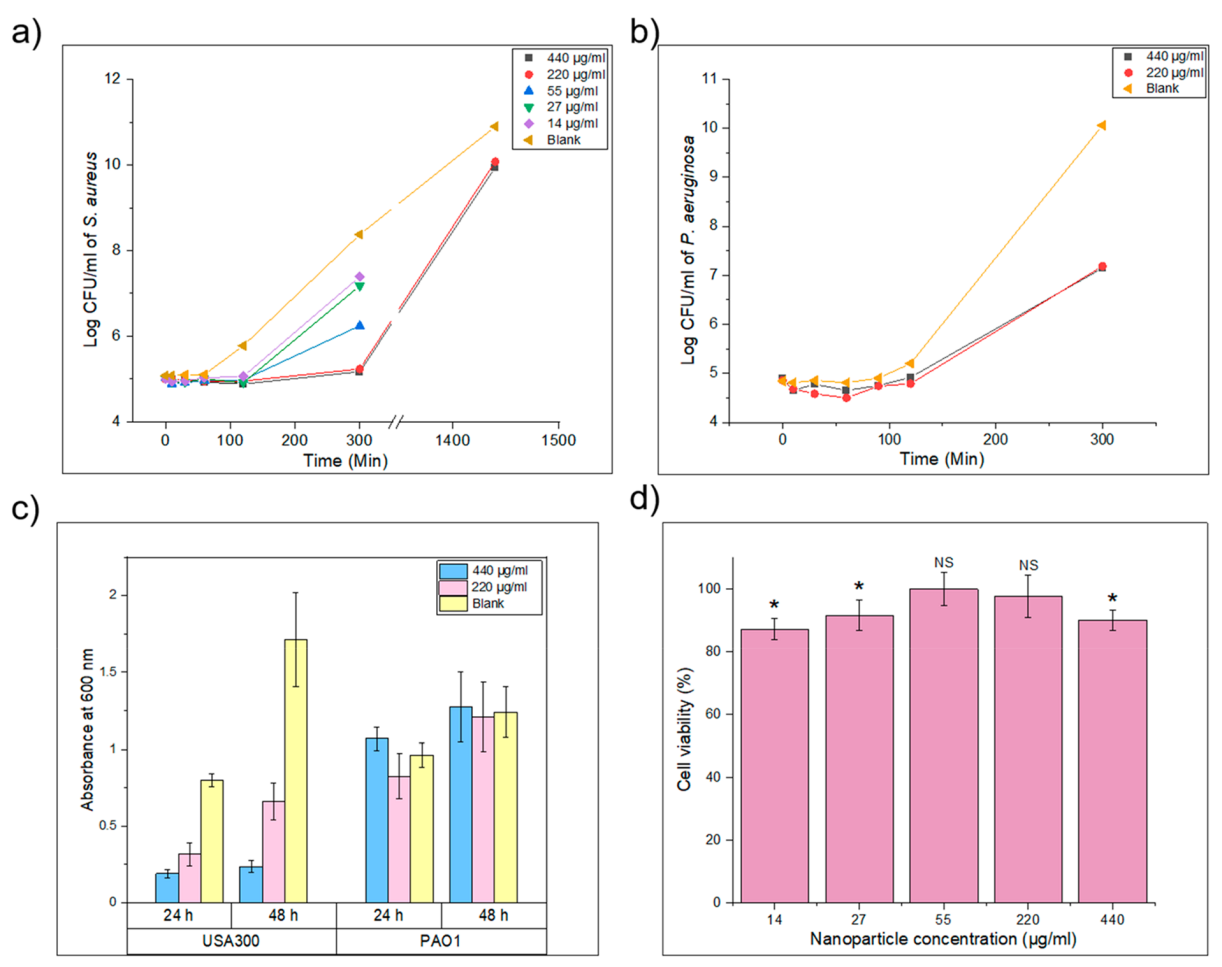
(a) *S. aureus* growth curve in the presence of S50-PA nanoparticles over 24 h in nutrient-rich LB medium. (b) *P. aeruginosa* growth curve in the presence of S50-PA nanoparticles after 5 h of incubation in nutrient-rich LB medium. (c) Absorbance at 600 nm after staining with crystal violet after 24 and 48 h of incubation at 37 °C with *S. aureus* (USA300) and *P. aeruginosa* (PAO1). (d) Cell viability (%) of HepG2 after treatment with S50-PA nanoparticles.

The antibacterial activity of bulk S50-PA has been reported previously; however, the antibacterial properties of nanoparticles of this material have not been reported.^14,15^ The antibacterial activity of S-polymer nanoparticles was assessed against Gram-positive methicillin-resistant *S. aureus* (strain USA300) and Gram-negative *P. aeruginosa* (strain PAO1). Nanoparticles were incubated in media containing bacteria. Viable cells were enumerated to assess whether the nanoparticles induce an antibacterial effect against the cells compared to a blank solution that was prepared by dropping THF into water and allowing the solution to stir overnight at room temperature. The preparation of the blank followed the same procedure as that used in the preparation of the polymer nanoparticles, however, with no polymer dissolved in the solvent phase. Polymer nanoparticles were tested at various concentrations in order to investigate the dose–response relationship. S50-PA nanoparticles at concentrations of 14, 27, 55, 220, and 440 μg/mL were tested over a period of 5 h (Figure 4a) against *S. aureus* in nutrient-rich LB medium, within which bacteria grow exponentially. After 5 h, a 1.07 (>90%) and 3.2 log (>99.9%) reduction in the number of viable cells compared to the blank was achieved for 14 and 440 μg/mL of nanoparticles, respectively. The higher concentrations of 220 and 440 μg/mL were further tested after 24 h and were found to have reduced the number of viable cells by 1.09 log (>90%), compared to the blank. The minimum inhibitory concentration (MIC) of S50-PA nanoparticles was evaluated during a 24 h incubation period with methicillin-resistant *S. aureus* (Figure S18). A 50% growth inhibition (MIC_50_) in *S. aureus,* compared to untreated samples, was found at a nanoparticle concentration of 64 μg/mL (*p* < 0.0001 relative to untreated control), and 90% inhibition (MIC_90_) was achieved at 512 μg/mL (*p* < 0.0001 relative to untreated control).

S50-Ger nanoparticles formulated in the same way were also found to have an inhibitory effect against *S. aureus* after a 5 h incubation period (Figure S19). When the initial *S. aureus* concentration was lowered from approximately 100,000 CFU/mL (5 log CFU/mL) to 100 CFU/mL (2 log CFU/mL) (Figure S20), a reduction in the number of viable cells following addition of nanoparticles was apparent, compared to the initial culture, suggesting that the S50-PA nanoparticles exert a bactericidal effect. That is, the particles kill bacterial cells, rather than merely suppressing cell growth. The effect of additional dosing of nanoparticles was also investigated (Figure S20); whereby the cultures were spiked with an additional 100 μL dose of particles or control after 2 h of incubation, the effect was enumearted after 5 h and it was found that the additional dose reduced the number of viable cells further.

S70-PA nanoparticles were also tested during an incubation period of 5 h against *S. aureus* in LB growth medium, with final nanoparticle concentrations of 14, 27, and 55 μg/mL (Figure S21). After 5 h of incubation, the same trend was observed whereby the higher the concentration of nanoparticles, the higher the log reduction in viable cells compared to the blank. At 14, 27, and 55 μg/mL of nanoparticles, a log reduction in viable cells of 3.31, 3.42, and 3.48 was achieved, respectively (>99.9% reduction in all cases). The log reduction for S50-PA nanoparticles at the same concentrations after 5 h were lower than those of S70-PA. This suggests that the S70-PA nanoparticles have an increased antibacterial effect compared to S50-PA nanoparticles against *S. aureus*. This could be due to the greater sulfur content withing S70-PA compared to S50-PA.

*P. aeruginosa* is a Gram-negative, rod shaped opportunistic pathogen that is a common cause of nosocomial infections and often shows innate resistance to a wide range of antibiotics.^27^ S50-PA polymeric nanoparticles were tested against *P. aeruginosa* strain PAO1 in nutrient-rich LB broth for a period of 5 h (Figure 4b) at final concentrations of 220 and 440 μg/mL. After 5 h of incubation, both concentrations of nanoparticles achieved a 2.9 log (>99%) reduction in viable *P. aeruginosa* cells compared to the control sample. In a 24 h assay, the MIC_50_ of S50-PA nanoparticles for *P. aeruginosa* PAO1was 128 μg/mL (*p* < 0.0001 relative to untreated control) (Figure S22). This shows that S50-PA polymeric nanoparticles behave similarly to the bulk material, which have been shown to exhibit an antibacterial effect against both *S. aureus* and *P. aeruginosa*.^15^ Similarly, S50-Ger nanoparticles were tested against *P. aeruginosa* PAO1 (Figure S23) and were also found to show an inhibitory effect, demonstrating that the antibacterial properties of the polymer nanoparticles are not restricted to only one type of comonomer used during polymer synthesis.

The potential of using high sulfur content nanoparticles as combination therapies with antibiotics was investigated. Nanoparticles were tested against an extensively drug-resistant *P. aeruginosa* strain (B9) in combination with tobramycin, an aminoglycoside antibiotic that is used to treat complicated infections such as septicaemia and urinary tract infections and to manage *P. aeruginosa* infections in people with cystic fibrosis.^28^ The effect of varying the tobramycin concentration (1–512 μg/mL) while maintaining a nanoparticle concentration of 128 μg/mL was investigated (Figure S24). In the absence of antibiotics, 512 μg/mL of nanoparticles were required to inhibit >50% of the growth (*p* < 0.0001 relative to untreated control) of the highly drug-resistant *P. aeruginosa* B9 strain. With treatment of tobramycin alone, B9 growth was completely inhibited only at the top concentration of 512 μg/mL (*p* < 0.0001 relative to untreated control); however, with the addition of nanoparticles, the growth was completely inhibited at a concentration of 256 μg/mL (*p* < 0.0001 relative to untreated control). Furthermore, reductions in growth with dual nanoparticle plus tobramycin treatment, relative to treatment with tobramycin alone, were observed at all tobramycin concentrations between 16 and 128 μg/mL. This demonstrates the potential of using the polymer nanoparticles in combination with other drugs. This may enable use of lower antibiotic concentrations, for antimicrobial stewardship, or help alleviate the side effects of long-term, high dose, antimicrobial therapy.

It is possible that, upon addition of the dissolved polymer into the antisolvent phase during the nanoprecipitation process, low molecular weight species could remain soluble in the antisolvent, i.e., water-soluble. To establish whether it is the nanoparticles that are having an antibacterial effect or if it is due to potential water-soluble species, a disc diffusion (Kirby-Bauer test) assay was conducted.^29^ In brief, empty antimicrobial susceptibility test discs were soaked with nanoparticles or water as the control. The loaded discs were placed on agar plates streaked with bacteria (*S. aureus* USA300 and *P. aeruginosa* PAO1) and incubated for 24 h. S50-PA nanoparticles were found to not have an antibacterial effect against *S. aureus* USA300 and *P. aeruginosa* PAO1 when tested using the disc diffusion assay (Figure S25). The results of the disc diffusion assay suggest that, if there are any low molecular weight, water-soluble species present, they are not responsible for the antibacterial activity observed. A possible reason S50-PA nanoparticles show reduced antibacterial activity on agar as compared to in liquid culture may be that the size of the particles limits or prohibits the diffusion of the nanoparticles through agar. Kourmoli et al. investigated the effect of particle size on the outcome of the disc diffusion assay.^29^ It was found that gold nanoparticles with diameters of 10–40 nm had negligible diffusibility through the agar and, therefore, did not exert an antimicrobial effect during the disc diffusion assay. S50-PA nanoparticles have been found to have z-average diameters of approximately 150 nm, determined by DLS and SEM (Figure 3), and are therefore larger than the Au nanoparticles investigated by Kourmoli et al.^29^ It is therefore plausible to suggest that S50-PA nanoparticles are too large to diffuse through the agar, and therefore are not able to exert an antibacterial effect during the disc diffusion method.^30^

High-sulfur content polymeric nanoparticles that are stable to high ionic strengths can be prepared by employing a nonionic surfactant in the nanoprecipitation (Figure S16). However, the presence of a nonionic surfactant could affect the antibacterial activity of S50-PA nanoparticles. The Tween80-stabilized particles were tested against *S. aureus* USA300 (Figure S26) and *P. aeruginosa* PAO1 (Figure S27) and were found to have a modestly enhanced inhibitory effect compared to S50-PA particles without Tween80. The charge of the polymer nanoparticles is expected to have an impact on the interaction with bacterial cells, for example, the lipopolysaccharide (LPS) component of Gram-negative outer membranes is negatively charged due to the presence of negatively charged phosphate groups.^30^ The ζ potential of S50-PA nanoparticles was found to be (−13.6 ± 1.2) mV when dispersed in LB medium, compared to (−47.5 ± 1.5) mV in water. Capping the nanoparticles with Tween80 was found to lower the magnitude of the negative ζ potential further (Figure S17). This may be the reason for the higher inhibitory effect of Tween80-capped particles against *S. aureus* compared to S50-PA particles without Tween80, due to a reduction in repulsive interactions between the negatively charged particles and the negatively charged cell wall of Gram-positive bacteria. We hypothesize that the antibacterial activity of the polymer nanoparticles can be tuned depending on the type of surfactant used. However, determining whether an increase or decrease in antibacterial activity with different surfactants is due to the charge differences alone, or, if the surfactant itself is having an effect would be difficult to determine. Therefore, instead of changing the charge of the nanoparticles, we conducted a study to modulate the charge of the bacterial surface, to probe the effect of charge interaction between nanoparticles and bacteria. For this study, we looked at the inhibition of two *P. aeruginosa* strains: LESB65 and LESB65Δ*pmrB* (Figure S28). The deletion of the *pmrB* gene in LESB65Δ*pmrB* results in a greater negative charge on the LPS of the outer membrane of the bacterial cell, as the absence of PmrB signaling restricts the activation of LPS modification pathways that add positively charged aminoarabinose to the lipid A component of LPS.^31^ Here, the nanoparticles showed an increased antibacterial effect against LESB65 compared to LESB65Δ*pmrB*, which may be due to increased repulsive charge interactions between LESB65Δ*pmrB* and the negatively charged nanoparticles. To further test this hypothesis, we preincubated both bacterial strains with the cationic polyamine spermidine. Preincubation with spermidine results in a less negatively charged outer membrane, as the polyamine coats the surface and negates the charge.^31^ The antibacterial activity of the nanoparticles against LESB65Δ*pmrB* was increased following preincubation with spermidine, as compared to in the absence of spermidine. This suggests that the charge interactions between the bacterial cells and nanoparticles are important to consider for future applications.

### Antibiofilm Activity

Biofilms provide favorable conditions for bacteria as they offer protection from the immune system of the host, exhibit phenotypic resistance to antimicrobial agents, and ensure efficient distribution of resources throughout the microbial population. These factors make biofilm bacteria more challenging than planktonic cells.^32^ The ability of S50-PA nanoparticles to inhibit biofilm formation on the surface of a container was evaluated against *S. aureus* USA300 and *P. aeruginosa* PAO1. Nanoparticles were added to vials containing bacterial culture and were statically incubated for 24 and 48 h; a control sample was prepared by adding water to the culture instead of nanoparticles. S50-PA nanoparticles were found to inhibit *S. aureus* biofilm formation over 48 h; however, the nanoparticles did not inhibit *P. aeruginosa* biofilm formation (Figure 4c). Sulfide containing molecules, such as allicin, have been reported to have much higher minimal inhibitory concentrations for *P. aeruginosa* in comparison to other organisms;^33^ therefore, the tested concentrations of nanoparticles in this instance may not have been high enough to achieve an inhibitory effect against *P. aeruginosa* over prolonged periods of time.

### Cytotoxicity

The cytotoxicity of high sulfur content polymers is not widely reported, and thus, their safety for uses in potential applications such as environmental remediation, IR optics, and novel antimicrobials is poorly understood. In 2016, Crockett et al. investigated the cytotoxicity of the product of the copolymerization of sulfur and limonene.^7^ The study found that water that has been exposed to the polysulfide was not cytotoxic during a 24 h contact time with the cells. The results are a promising start to investigating the safety of high sulfur content polymers.^7^ It is expected that the comonomers used during the copolymerization of elemental sulfur will result in materials with different properties and perhaps cytotoxicities. Furthermore, the properties of a nanomaterial can often differ from the bulk due to an increase in surface area. The cytotoxicity of high sulfur content polymeric nanoparticles against the liver carcinoma cell line HepG2 was investigated using the MTT assay.^34^

In brief, the nanoparticles were added to approximately 80% confluent HepG2 cell cultures and were incubated for 24 h. Untreated cells were used as the negative control. After incubation, the culture media was removed and replaced with fresh medium containing MTT labeling agent. The solutions were incubated for 4 h, before the solubilizing agent was added. The absorbance at 595 nm was measured after leaving the solutions in the presence of the solubilizing agent to incubate overnight. S50-PA nanoparticles at final concentrations of 14, 27, 55, 220, and 440 μg/mL were evaluated for their cytotoxicity against HepG2 cells. The cytotoxicity of the nanoparticles was expressed as % cell viability compared to untreated HepG2 cells (Figure 4d). More than 80% cell viability was observed at all nanoparticle concentrations tested, and no clear dose-dependent toxicity was observed.

### Cysteine Mediated H_2_S Release

**Figure 5:**
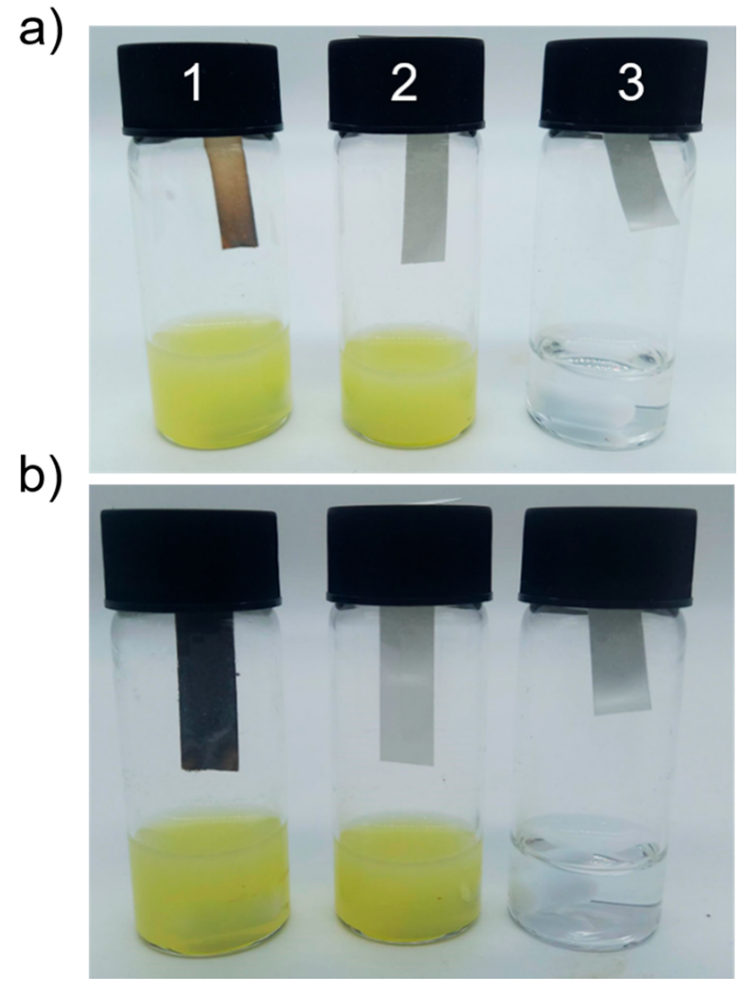
(a) Lead acetate paper after 5 min of exposure and (b) 5 h of exposure to (1) S50-PA nanoparticles and cysteine, (2) S50-PA nanoparticles, and (3) cysteine.

**Figure 6:**
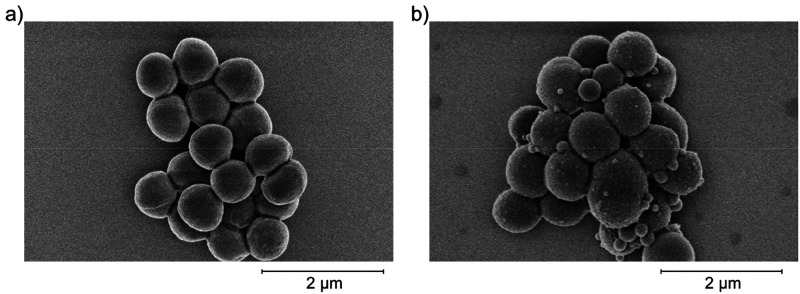
SEM images of *S. aureus* (a) before and (b) after treatment with S50-PA nanoparticles.

The mechanism of action of high-sulfur content polymers against bacteria is poorly understood. Many studies have been conducted into the antimicrobial activity of allyl sulfides such as diallyl sulfide, diallyl disulfide, and diallyl trisulfide, and several possible mechanisms of action have been postulated, one of which is the interaction of polysulfides with thiol-containing enzymes and membranes of bacterial cells.^35−38^ It has been reported that sulfides can undergo exchange reactions with thiols, such as cysteine, forming H_2_S as a byproduct of the exchange.^39,40^ To probe whether S50-PA nanoparticles can interact with cellular thiols, cysteine was chosen as a model thiol. In brief, cysteine was added to S50-PA nanoparticles diluted in water under stirring. The generation of H_2_S was assessed qualitatively using lead(II) acetate test strips (lower detection limit of 4 ppm of H_2_S) adhered to the inside of the cap of the vial (Figure 5). Two controls were prepared: one consisted of cysteine in the absence of S50-PA nanoparticles and the other contained S50-PA nanoparticles in the absence of cysteine. After 5 min of stirring at room temperature, a color change in the lead(II) acetate test strip was observed for the vial containing S50-PA nanoparticles and cysteine. The brown color is consistent with that of lead sulfide, suggesting that H_2_S was generated in this vial during the first 5 min of stirring. After 5 h, the color of the strip darkened further. No color change was observed during the 5 h period for either of the control samples. H_2_S generation during the incubation of *S. aureus* with S50-PA nanoparticles was investigated during a 24 h period (Figure S29). During the 24 h incubation period, it was found that H2S was generated in the vial containing nanoparticles and *S. aureus*; no H_2_S was detected in the controls. The results show that S50-PA nanoparticles are able to form H_2_S in the presence of cysteine and *S. aureus* cells, which suggests that the particles are able to undergo exchange reactions with cellular thiols. SEM imaging of *S. aureus* cells before and after treatment with S50-PA nanoparticles (Figure 6) shows the presence of smaller particles, consistent with the morphologies observed in the SEM images of the nanoparticles, on the surface of the bacterial cells. The bacterial cells lost uniformity of size and shape following nanoparticle treatment. It is therefore possible that the particles are exerting an effect by directly interacting with the surface of the cells.

## Conclusions

In summary, we have shown that high sulfur content polymeric nanoparticles can be prepared by both nanoprecipitation and emulsion-templated methods. The nanoparticles are water-dispersible, with average hydrodynamic radii of approximately 100–200 nm. High sulfur content polymeric nanoparticles show antibacterial activity against both Gram-positive methicillin-resistant *S. aureus* and Gram-negative *P. aeruginosa* and were shown to inhibit *S. aureus* biofilm formation over 48 h. Particles were found to be instable in the presence of salts. However, stabilized particles could be formed by the addition of a surfactant such as Tween80, which does not inhibit the antibacterial activity of the particles. This study also sheds important light on the potential mechanism of action of sulfur polymers, which needs to be better understood. One possible mechanism could be interaction with cellular thiols, as demonstrated by the interaction of the nanoparticles with cysteine. S50-PA polymeric nanoparticles show little cytotoxicity against the HepG2 cell line at various concentrations spanning 14–440 μg/mL. The results show that high sulfur content polymeric nanoparticles could have potential biomedical applications such as for wound dressings or catheter coatings; however, the water-dispersible nanoparticles could find applications in other fields such as delaying bacterial colonization of standing water or for environmental remediation.

## Supporting Information Available

Figures of photograph of the S50-Ger polymer in a range of solvents, chemical structures of the surfactants investigated for the preparation of polymer nanoparticles, image of the aqueous dispersion of S50-Ger formed by an emulsion/solvent evaporation method, size distribution by intensity and correlogram traces, summary of the z-average diameter and PDI, images of the chloroform in water images, DLS traces of S50-PA nanoparticles, ζ potential of S50-PA nanoparticles, graph summarizing the % growth of *S. aureus* relative to a positive control, *S. aureus* growth curves, graph summarizing the % growth of *P. aeruginosa* relative to a positive control, *P. aeruginosa* growth curves, image of agar plate streaked with *S. aureus* USA 300 and *P. aeruginosa* PAO1, log increase in LESB65 and *ΔpmrB* compared to initial culture, and lead acetate paper after 24 h of exposure and table of calculated and obtained elemental analysis of sulfur-geraniol polysulfides (PDF)

## Abbreviations Used

DLS: dynamic light scattering
SEM: scanning electron microscopy
NMR: nuclear magnetic resonance
FTIR: Fourier transform infrared
PDI: polydispersity index

## References

[ref4] Chung W. J.; Griebel J. J.; Kim E. T.; Yoon H.; Simmonds A. G.; Ji H. J.; Dirlam P. T.; Glass R. S.; Wie J. J.; Nguyen N. A.; Guralnick B. W.; Park J.; Somogyi Á.; Theato P.; Mackay M. E.; Sung Y.-E.; Char K.; Pyun J. The Use of Elemental Sulfur as an Alternative Feedstock for Polymeric Materials. Nat. Chem. 2013, 5 (6), 518–524. 10.1038/nchem.1624. 23695634

[ref2] Gomez I.; Mecerreyes D.; Blazquez J. A.; Leonet O.; Ben Youcef H.; Li C.; Gómez-Cámer J. L.; Bondarchuk O.; Rodriguez-Martinez L. Inverse Vulcanization of Sulfur with Divinylbenzene: Stable and Easy Processable Cathode Material for Lithium-Sulfur Batteries. J. Power Sources 2016, 329, 72–78. 10.1016/j.jpowsour.2016.08.046.

[ref3] Smith J. A.; Green S. J.; Petcher S.; Parker D. J.; Zhang B.; Worthington M. J. H.; Wu X.; Kelly C. A.; Baker T.; Gibson C. T.; Campbell J. A.; Lewis D. A.; Jenkins M. J.; Willcock H.; Chalker J. M.; Hasell T. Crosslinker Copolymerization for Property Control in Inverse Vulcanization. Chem. Eur. J. 2019, 25 (44), 10433–10440. 10.1002/chem.201901619. 31136036

[ref5] Hoefling A.; Lee Y. J.; Theato P. Sulfur-Based Polymer Composites from Vegetable Oils and Elemental Sulfur: A Sustainable Active Material for Li–S Batteries. Macromol. Chem. Phys. 2017, 218 (1), 1600303 10.1002/macp.201600303.

[ref6] Parker D. J.; Chong S. T.; Hasell T. Sustainable Inverse-Vulcanised Sulfur Polymers. RSC Adv. 2018, 8 (49), 27892–27899. 10.1039/C8RA04446E. 35542731 PMC9083557

[ref7] Crockett M. P.; Evans A. M.; Worthington M. J. H.; Albuquerque I. S.; Slattery A. D.; Gibson C. T.; Campbell J. A.; Lewis D. A.; Bernardes G. J. L.; Chalker J. M. Sulfur-Limonene Polysulfide: A Material Synthesized Entirely from Industrial By-Products and Its Use in Removing Toxic Metals from Water and Soil. Angew. Chem., Int. Ed. Engl. 2016, 55 (5), 1714–1718. 10.1002/anie.201508708. 26481099 PMC4755153

[ref8] Worthington M. J. H.; Shearer C. J.; Esdaile L. J.; Campbell J. A.; Gibson C. T.; Legg S. K.; Yin Y.; Lundquist N. A.; Gascooke J. R.; Albuquerque I. S.; Shapter J. G.; Andersson G. G.; Lewis D. A.; Bernardes G. J. L.; Chalker J. M. Sustainable Polysulfides for Oil Spill Remediation: Repurposing Industrial Waste for Environmental Benefit. Adv. Sustain. Syst. 2018, 2 (6), 1800024 10.1002/adsu.201800024.

[ref9] Griebel J. J.; Nguyen N. A.; Namnabat S.; Anderson L. E.; Glass R. S.; Norwood R. A.; Mackay M. E.; Char K.; Pyun J. Dynamic Covalent Polymers via Inverse Vulcanization of Elemental Sulfur for Healable Infrared Optical Materials. ACS Macro Lett. 2015, 4 (9), 862–866. 10.1021/acsmacrolett.5b00502. 35596448

[ref10] Valle S. F.; Giroto A. S.; Klaic R.; Guimarães G. G. F.; Ribeiro C. Sulfur Fertilizer Based on Inverse Vulcanization Process with Soybean Oil. Polym. Degrad. Stab. 2019, 162, 102–105. 10.1016/j.polymdegradstab.2019.02.011.

[ref1] Deng Z.; Hoefling A.; Théato P.; Lienkamp K. Surface Properties and Antimicrobial Activity of Poly(Sulfur-Co-1,3-Diisopropenylbenzene) Copolymers. Macromol. Chem. Phys. 2018, 219 (5), 1700497 10.1002/macp.201700497.

[ref11] Cubero-Cardoso J.; Gómez-Villegas P.; Santos-Martín M.; Sayago A.; Fernández-Recamales Á.; Fernández de Villarán R.; Cuadri A. A.; Martín-Alfonso J. E.; Borja R.; Fermoso F. G.; León R.; Urbano J. Combining Vegetable Oils and Bioactive Compounds via Inverse Vulcanization for Antioxidant and Antimicrobial Materials. Polym. Test. 2022, 109, 107546 10.1016/j.polymertesting.2022.107546.

[ref12] Upton R. L.; Dop R. A.; Sadler E.; Lunt A. M.; Neill D. R.; Hasell T.; Crick C. R. Investigating the Viability of Sulfur Polymers for the Fabrication of Photoactive, Antimicrobial, Water Repellent Coatings. J. Mater. Chem. B 2022, 10 (22), 4153–4162. 10.1039/D2TB00319H. 35438120

[ref13] Lim J.; Jung U.; Joe W. T.; Kim E. T.; Pyun J.; Char K. High Sulfur Content Polymer Nanoparticles Obtained from Interfacial Polymerization of Sodium Polysulfide and 1,2,3-Trichloropropane in Water. Macromol. Rapid Commun. 2015, 36 (11), 1103–1107. 10.1002/marc.201500006. 25847485

[ref14] Zhang B.; Petcher S.; Dop R. A.; Yan P.; Zhao W.; Wang H.; Dodd L. J.; McDonald T. O.; Hasell T. Inverse Vulcanised Sulfur Polymer Nanoparticles Prepared by Antisolvent Precipitation. J. Mater. Chem. A 2022, 10 (26), 13704–13710. 10.1039/D2TA01653B.

[ref15] Dop R. A.; Neill D. R.; Hasell T. Antibacterial Activity of Inverse Vulcanized Polymers. Biomacromolecules 2021, 22 (12), 5223–5233. 10.1021/acs.biomac.1c01138. 34784205 PMC7614836

[ref16] EUCAST reading guide for broth microdilution, version 4.0; European Committee on Antimicrobial Susceptibility Testing, 2022.

[ref17] Cazares A.; Moore M. P.; Hall J. P. J.; Wright L. L.; Grimes M.; Emond-Rhéault J.-G.; Pongchaikul P.; Santanirand P.; Levesque R. C.; Fothergill J. L.; Winstanley C. A Megaplasmid Family Driving Dissemination of Multidrug Resistance in Pseudomonas. Nat. Commun. 2020, 11 (1), 1370 10.1038/s41467-020-15081-7. 32170080 PMC7070040

[ref18] Ali B.; Al-Wabel N. A.; Shams S.; Ahamad A.; Khan S. A.; Anwar F. Essential Oils Used in Aromatherapy: A Systemic Review. Asian Pac. J. Trop. Biomed. 2015, 5 (8), 601–611. 10.1016/j.apjtb.2015.05.007.

[ref19] Chen T. C.; Fonseca C. O. Da; Schönthal A. H. Preclinical Development and Clinical Use of Perillyl Alcohol for Chemoprevention and Cancer Therapy. Am. J. Cancer Res. 2015, 5 (5), 1580–1593. 26175929 PMC4497427

[ref20] Maladeniya C.; Karunarathna M.; Lauer M.; Lopez C.; Thiounn T.; Smith R. A Role for Terpenoid Cyclization in the Atom Economical Polymerization of Terpenoids with Sulfur to Yield Durable Composites. Mater. Adv. 2020, 1 (6), 1665 10.1039/D0MA00474J.

[ref21] Quérette T.; Bordes C.; Sintes-Zydowicz N. Non-Isocyanate Polyurethane Nanoprecipitation: Toward an Optimized Preparation of Poly(Hydroxy)Urethane Nanoparticles. Colloids Surfaces A Physicochem. Eng. Asp. 2020, 589, 124371 10.1016/j.colsurfa.2019.124371.

[ref22] Guhagarkar S.; Malshe V.; Devarajan P. Nanoparticles of Polyethylene Sebacate: A New Biodegradable Polymer. AAPS PharmSciTech 2009, 10 (3), 935–942. 10.1208/s12249-009-9284-4. 19629708 PMC2802147

[ref23] Zhang X.; Servos M. R.; Liu J. Ultrahigh Nanoparticle Stability against Salt, PH, and Solvent with Retained Surface Accessibility via Depletion Stabilization. J. Am. Chem. Soc. 2012, 134 (24), 9910–9913. 10.1021/ja303787e. 22646098

[ref24] Cortés H.; Hernández-Parra H.; Bernal-Chávez S. A.; Prado-Audelo M. L. D.; Caballero-Florán I. H.; Borbolla-Jiménez F. V.; González-Torres M.; Magaña J. J.; Leyva-Gómez G. Non-Ionic Surfactants for Stabilization of Polymeric Nanoparticles for Biomedical Uses. Materials 2021, 14 (12), 3197 10.3390/ma14123197. 34200640 PMC8226872

[ref25] Felicia L.; Johnson J.; Philip J. Effect of Surfactant on the Size, Zeta Potential and Rheology of Alumina Nanofluids. J. Nanofluids 2014, 3 (4), 328 10.1166/jon.2014.1119.

[ref26] Choi W.; Mahajan U.; Lee S.-M.; Abiade J.; Singh R. K. Effect of Slurry Ionic Salts at Dielectric Silica CMP. J. Electrochem. Soc. 2004, 151 (3), G185 10.1149/1.1644609.

[ref27] Fazeli H.; Akbari R.; Moghim S.; Narimani T.; Arabestani M. R.; Ghoddousi A. R. Pseudomonas Aeruginosa Infections in Patients, Hospital Means, and Personnel’s Specimens. J. Res. Med. Sci. 2012, 17 (4), 332–337. 23267393 PMC3526125

[ref28] Rosalia M.; Chiesa E.; Tottoli E. M.; Dorati R.; Genta I.; Conti B.; Pisani S. Tobramycin Nanoantibiotics and Their Advantages: A Minireview. Int. J. Mol. Sci. 2022, 23 (22), 14080 10.3390/ijms232214080. 36430555 PMC9692674

[ref29] Kourmouli A.; Valenti M.; van Rijn E.; Beaumont H. J. E.; Kalantzi O.-I.; Schmidt-Ott A.; Biskos G. Can Disc Diffusion Susceptibility Tests Assess the Antimicrobial Activity of Engineered Nanoparticles?. J. Nanoparticle Res. 2018, 20 (3), 62 10.1007/s11051-018-4152-3. PMC5834581 29527123

[ref30] Bertani B.; Ruiz N. Function and Biogenesis of Lipopolysaccharides. EcoSal Plus 2018, 8 (1), 1 10.1128/ecosalplus.ESP-0001-2018. PMC6091223 30066669

[ref31] Hasan C. M.; Pottenger S.; Green A. E.; Cox A. A.; White J. S.; Jones T.; Winstanley C.; Kadioglu A.; Wright M. H.; Neill D. R.; Fothergill J. L. Pseudomonas Aeruginosa Utilizes the Host-Derived Polyamine Spermidine to Facilitate Antimicrobial Tolerance. JCI Insight 2022, 7 (22), e158879 10.1172/jci.insight.158879. 36194492 PMC9746822

[ref32] Tasse J.; Trouillet-Assant S.; Josse J.; Martins-Simões P.; Valour F.; Langlois-Jacques C.; Badel-Berchoux S.; Provot C.; Bernardi T.; Ferry T.; Laurent F. Association between Biofilm Formation Phenotype and Clonal Lineage in Staphylococcus Aureus Strains from Bone and Joint Infections. PLoS One 2018, 13 (8), e0200064 10.1371/journal.pone.0200064. 30161132 PMC6116976

[ref33] Müller A.; Eller J.; Albrecht F.; Prochnow P.; Kuhlmann K.; Bandow J. E.; Slusarenko A. J.; Leichert L. I. O. Allicin Induces Thiol Stress in Bacteria through S-Allylmercapto Modification of Protein Cysteines. J. Biol. Chem. 2016, 291 (22), 11477–11490. 10.1074/jbc.M115.702308. 27008862 PMC4882420

[ref34] Ghasemi M.; Turnbull T.; Sebastian S.; Kempson I. The MTT Assay: Utility, Limitations, Pitfalls, and Interpretation in Bulk and Single-Cell Analysis. Int. J. Mol. Sci. 2021, 22 (23), 12827 10.3390/ijms222312827. 34884632 PMC8657538

[ref35] Li W.-R.; Ma Y.-K.; Xie X.-B.; Shi Q.-S.; Wen X.; Sun T.-L.; Peng H. Diallyl Disulfide From Garlic Oil Inhibits Pseudomonas Aeruginosa Quorum Sensing Systems and Corresponding Virulence Factors. Front. Microbiol. 2019, 9, 3222 10.3389/fmicb.2018.03222. 30666240 PMC6330763

[ref36] Kim J.; Huh J.; Kyung S.; Kyung K. Antimicrobial Activity of Alk (En) Yl Sulfides Found in Essential Oils of Garlic and Onion. Food Sci. Biotechnol. 2004, 13 (2), 235–239.

[ref37] Ross Z. M.; O’Gara E. A.; Hill D. J.; Sleightholme H. V.; Maslin D. J. Antimicrobial Properties of Garlic Oil against Human Enteric Bacteria: Evaluation of Methodologies and Comparisons with Garlic Oil Sulfides and Garlic Powder. Appl. Environ. Microbiol. 2001, 67 (1), 475–480. 10.1128/AEM.67.1.475-480.2001. 11133485 PMC92605

[ref38] Münchberg U.; Anwar A.; Mecklenburg S.; Jacob C. Polysulfides as Biologically Active Ingredients of Garlic. Org. Biomol. Chem. 2007, 5 (10), 1505–1518. 10.1039/B703832A. 17571177

[ref39] Liang D.; Wu H.; Wong M. W.; Huang D. Diallyl Trisulfide Is a Fast H2S Donor, but Diallyl Disulfide Is a Slow One: The Reaction Pathways and Intermediates of Glutathione with Polysulfides. Org. Lett. 2015, 17 (17), 4196–4199. 10.1021/acs.orglett.5b01962. 26301500

[ref40] Cerda M. M.; Hammers M. D.; Earp M. S.; Zakharov L. N.; Pluth M. D. Applications of Synthetic Organic Tetrasulfides as H2S Donors. Org. Lett. 2017, 19 (9), 2314–2317. 10.1021/acs.orglett.7b00858. 28440074 PMC6022400

