# Supplementary material for: Sulfur-Polymer Nanoparticles: Preparation and Antibacterial Activity
Source: ACS Appl Mater Interfaces. 2023 Apr 19;15(17):20822–32. doi: 10.1021/acsami.3c03826 (PMC10165599; doi:10.1021/acsami.3c03826)
Supplement: Supplementary file 1 — am3c03826_si_001.pdf [file am3c03826_si_001.pdf]

Electronic Supplementary Information:

Sulfur-polymer Nanoparticles: Preparation and Antibacterial Activity

Romy A. Dop[1,2], Daniel R. Neill[2*] and Tom Hasell[1*]*

1 Department of Chemistry, University of Liverpool, Liverpool L69 7ZD, United Kingdom

2 Department of Clinical Infection, Microbiology and Immunology, Institute of Infection, Veterinary and Ecological Sciences, University of Liverpool, Liverpool L69 7ZD, United Kingdom

3 College of Chemistry and Chemical Engineering, Gansu International Scientific and Technological Cooperation Base of Water-Retention Chemical Functional Materials, Northwest Normal University, Lanzhou 730070, P. R. China

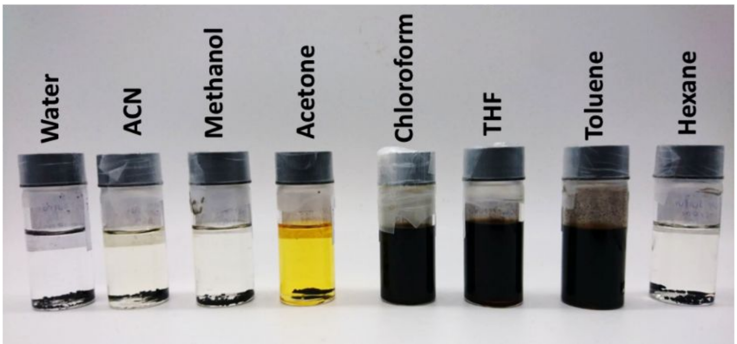

Figure S1: Photograph of the S50-Ger polymer in a range of solvents

Table S1: Calculated and obtained elemental analysis of sulfur-geraniol polysulfides.

**Sample**	Calc. % Carbon	**Calc. % Hydrogen**	Calc. % Sulfur	**C/H ratio**	Actual % Carbon	**Actual % Hydrogen**	Actual % Sulfur	**C/H ratio**
**S-geraniol 50:50**	38.94	**5.88**	50	**6.62**	42.76	**5.65**	49.28	**7.57**
**S-geraniol 70:30**	23.36	**3.53**	70	**6.62**	23.08	**3.20**	72.97	**7.22**

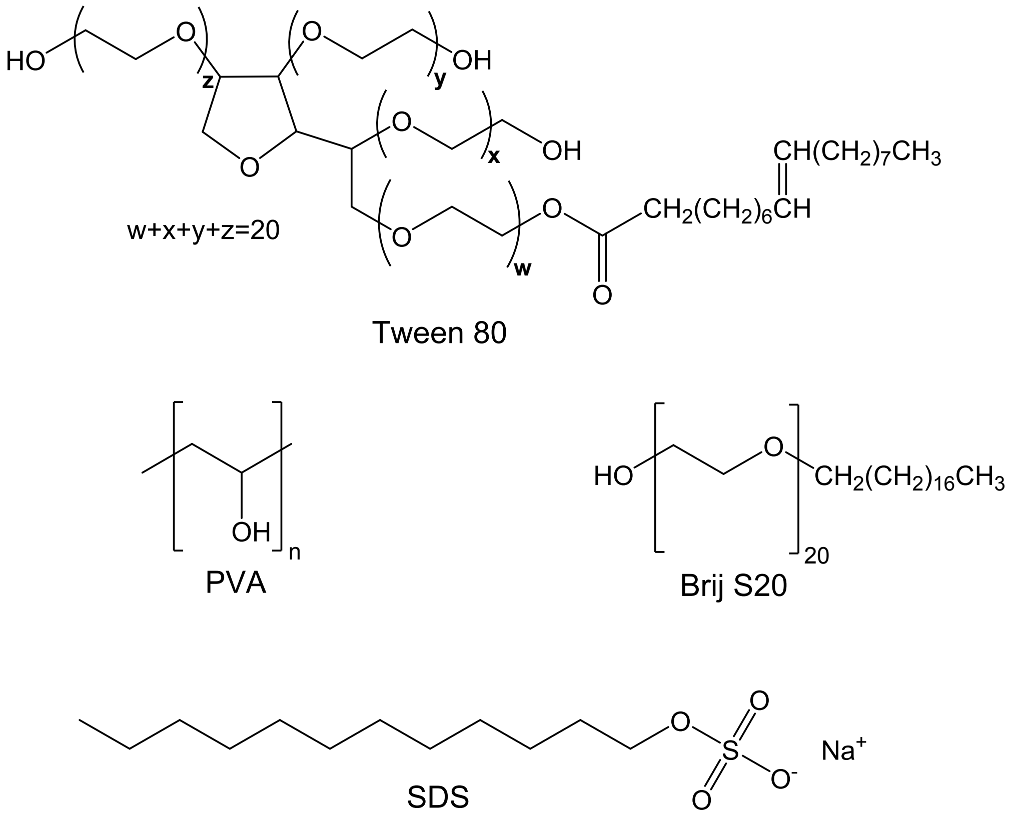

Figure S2: The chemical structures of the surfactants investigated for the preparation of polymer nanoparticles

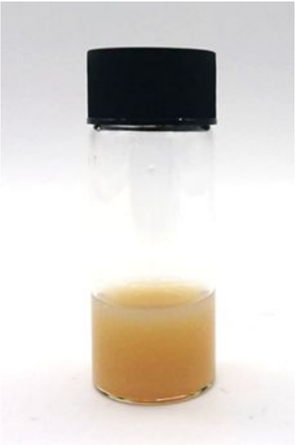

Figure S3: Image of the aqueous dispersion of S50-Ger formed by an emulsion/solvent evaporation method.

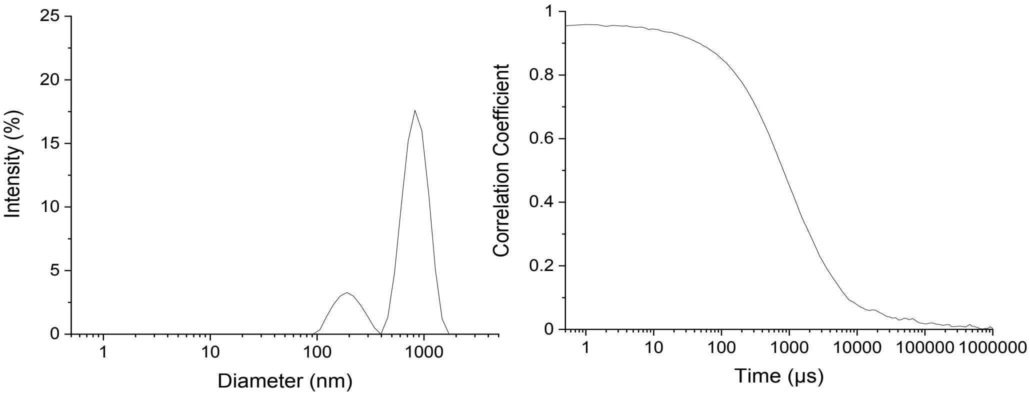

Figure S4: The size distribution by intensity and correlogram traces obtained for dispersions of S50-Ger formed by an emulsion-solvent evaporation method employing Brij S20 as a surfactant.

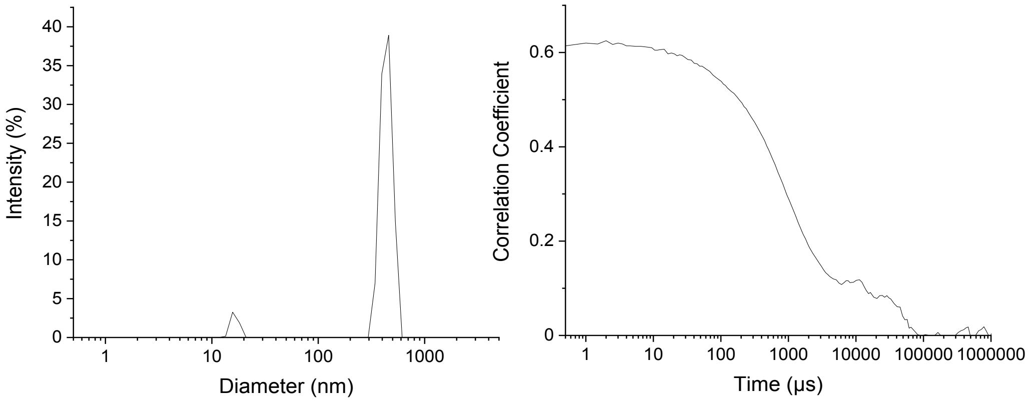

Figure S5: The size distribution by intensity and correlogram traces obtained for dispersions S50-Ger formed by an emulsion-solvent evaporation method employing SDS as a surfactant.

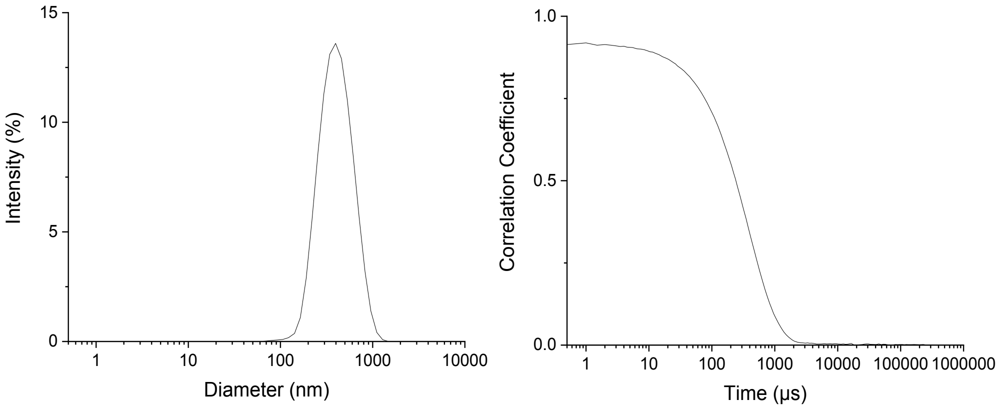

Figure S6: The size distribution by intensity and correlogram traces obtained for dispersions of sulfur-geraniol (50:50) polysulfide formed by an emulsion-solvent evaporation method employing PVA as a surfactant.

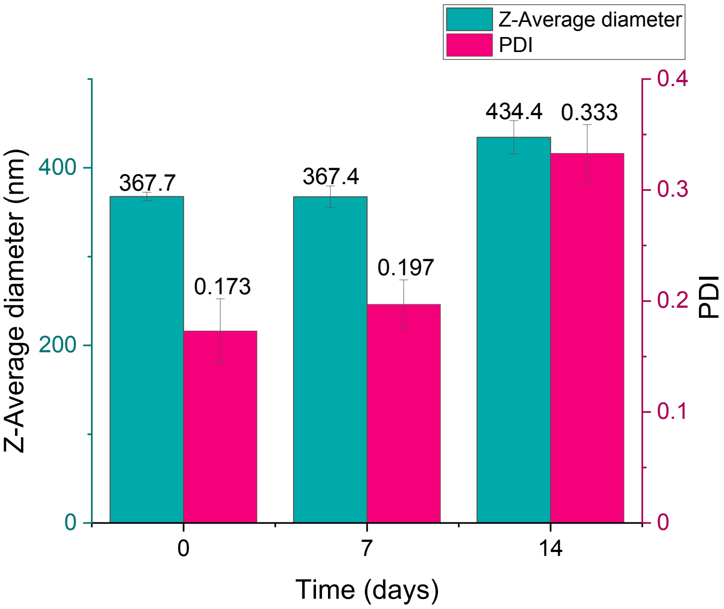

Figure S7: A summary of the z-average diameter and PDI obtained for dispersions of S50-Ger formed by an emulsion-solvent evaporation method with PVA after 0, 7 and 14 days.

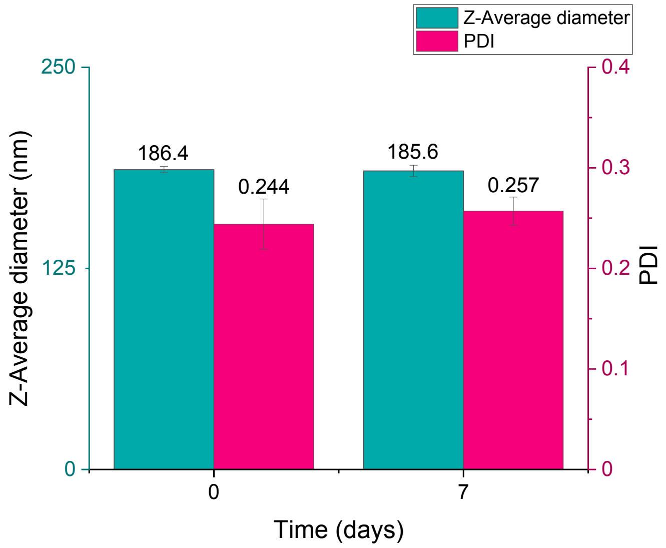

Figure S8: A summary of the z-average diameter and PDI values obtained for dispersions of S50-Ger formed by an emulsion-solvent evaporation method with Tween 80 after 0 and 7 days

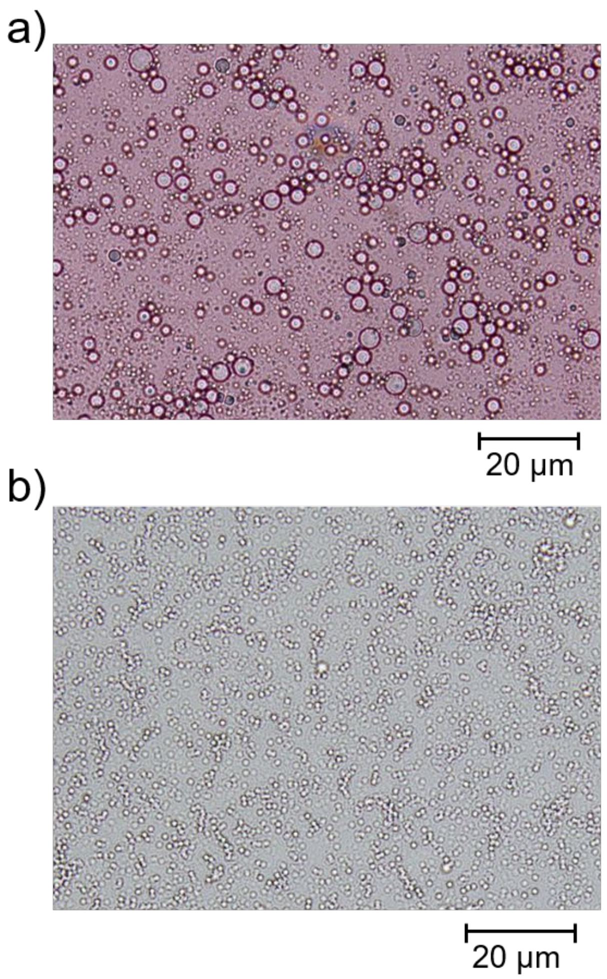

Figure S9: Images of the chloroform in water images with a) PVA and b) Tween80 as surfactants.

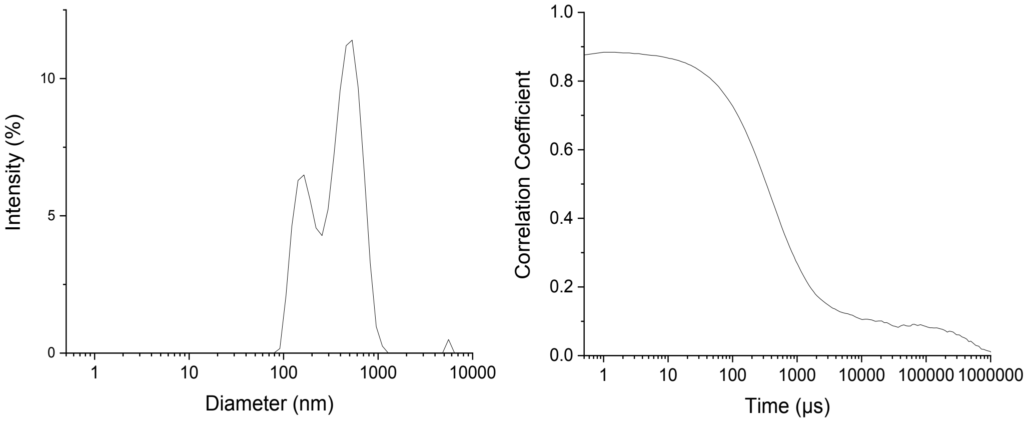

Figure S10: The size distribution by intensity and correlogram traces obtained for dispersions of S50-Ger formed by an emulsion-solvent evaporation method employing Tween 80 at a concentration of 0.05 mg/ml.

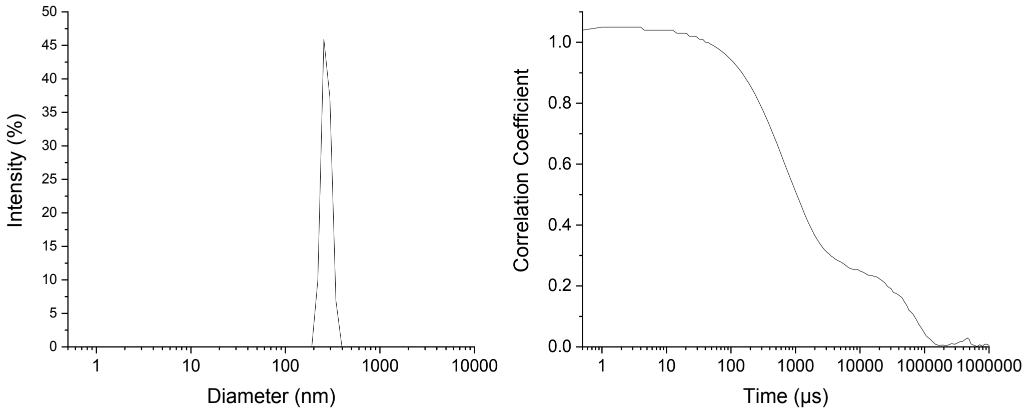

Figure S11: The size distribution by intensity and correlogram traces obtained for dispersions of S50-Ger formed by an emulsion-solvent evaporation method employing Tween 80 at a concentration of 0.1 mg/ml.

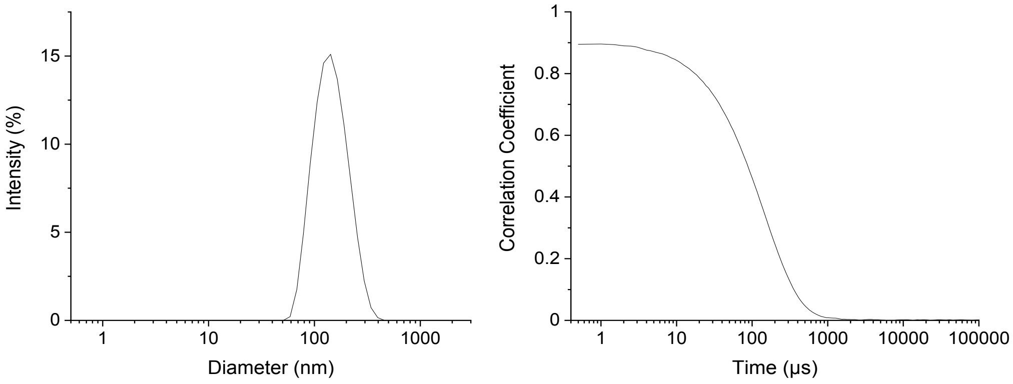

Figure S12: The size distribution by intensity and correlogram traces obtained for dispersions of S50-Ger formed by a nanoprecipitation method without a surfactant.

.

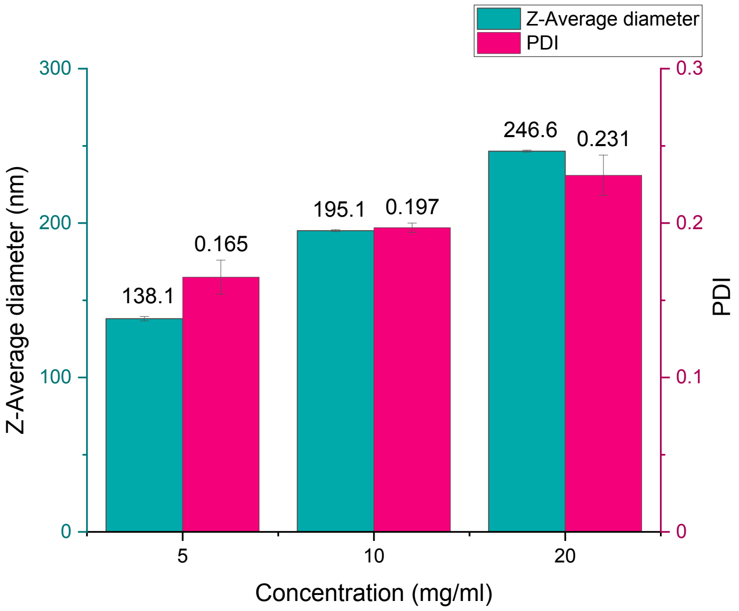

Figure S13: A summary of the z-average diameter and PDI values obtained for a dispersion of S50-Ger formed by a nanoprecipitation method without surfactant at varying polymer concentrations.

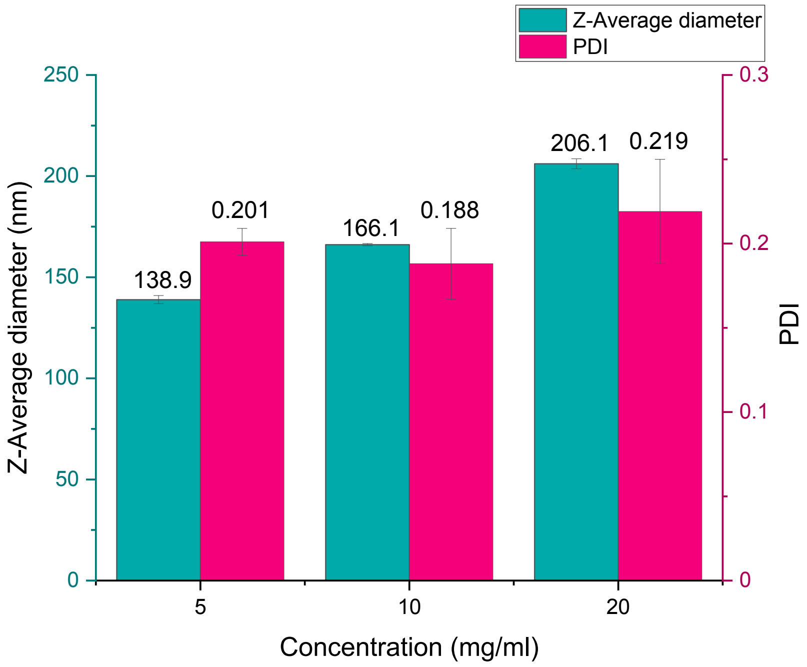

Figure S14: A summary of the z-average diameter and PDI values obtained for a dispersion of S50-Ger formed by a nanoprecipitation method with Tween 80 at varying polymer concentrations.

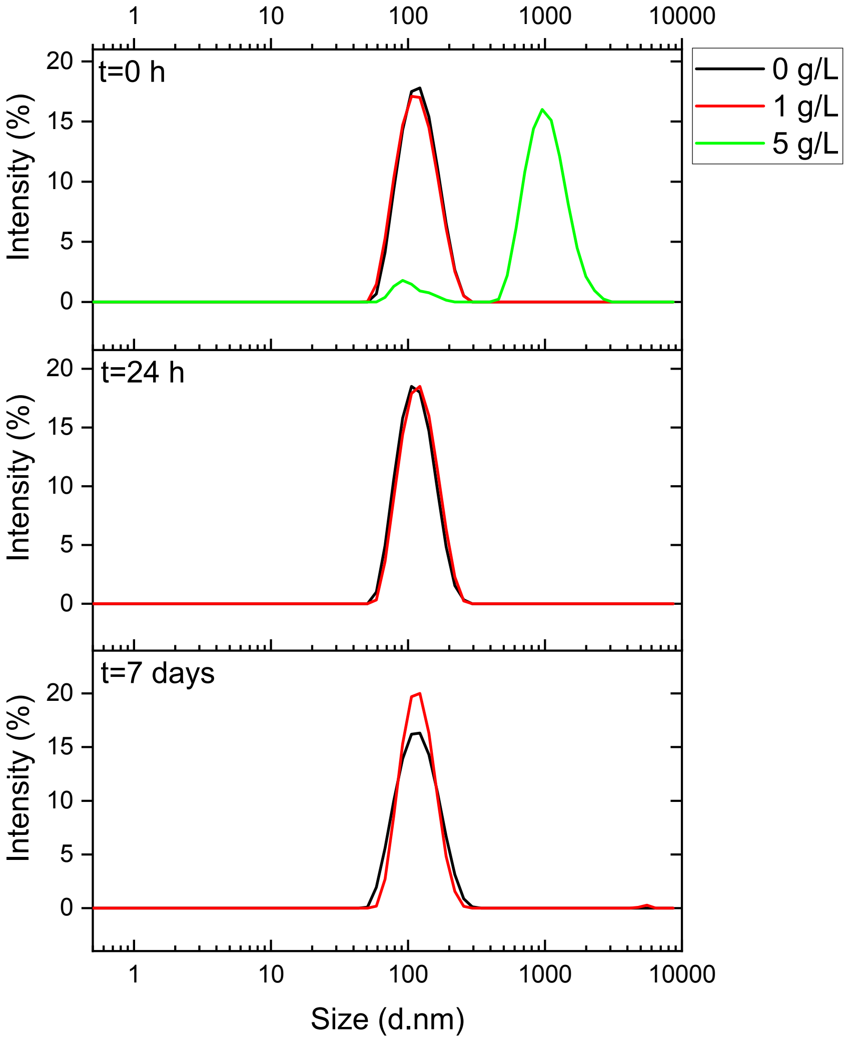

Figure S15: DLS traces of S50-PA nanoparticles in water in the presence of NaCl at various concentrations (0,1, 5, 25, 50 and 100 g/L) after 0, 1 and 7 days.

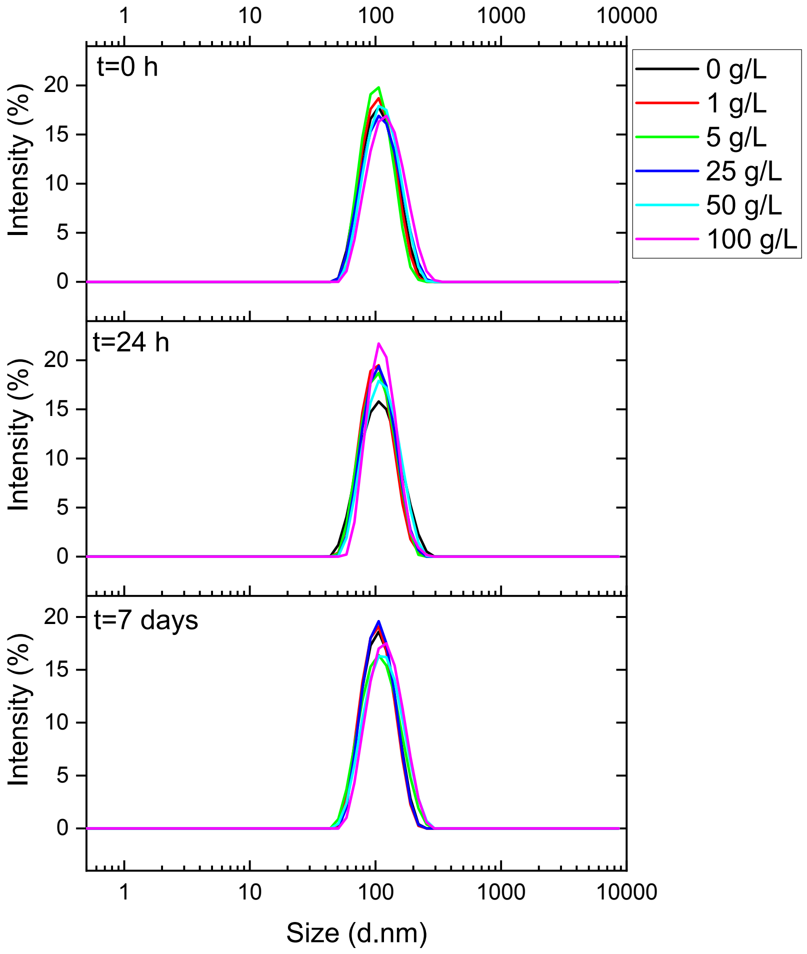

Figure S16: DLS traces of S50-PA nanoparticles in Tween80 in the presence of NaCl at various concentrations (0,1, 5, 25, 50 and 100 g/L) after 0, 1 and 7 days.

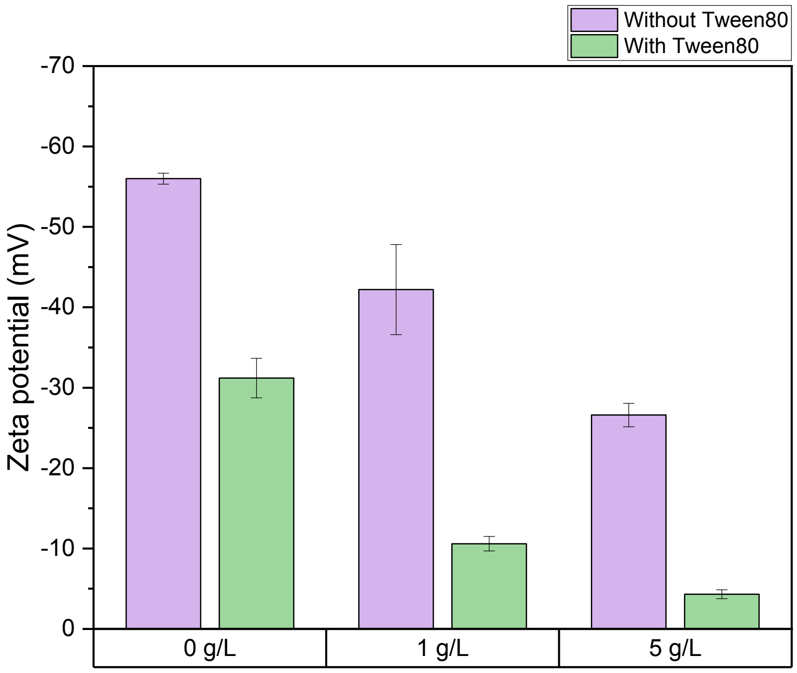

Figure S17:The zeta potential of S50-PA nanoparticles forumlated with and without Tween80 in the presence of NaCL at 0, 1 and 5 g/L.

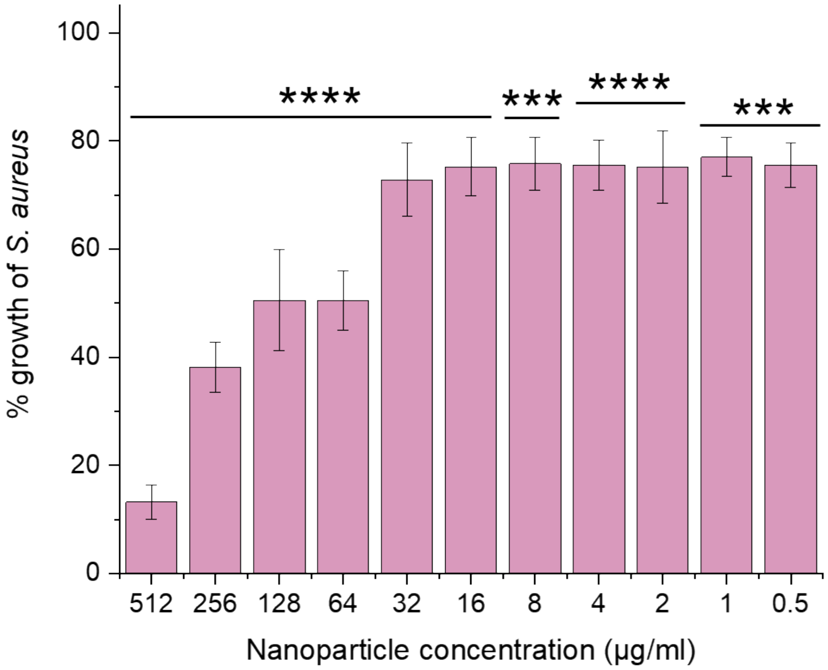

Figure S18: Graph summarising the % growth of *S. aureus* relative to a positive control, in the presence of S50-PA nanoparticles at various concentrations during a 24 h incubation period. ***$p <$ 0.001, ****$p <$ 0.0001 compared to control.

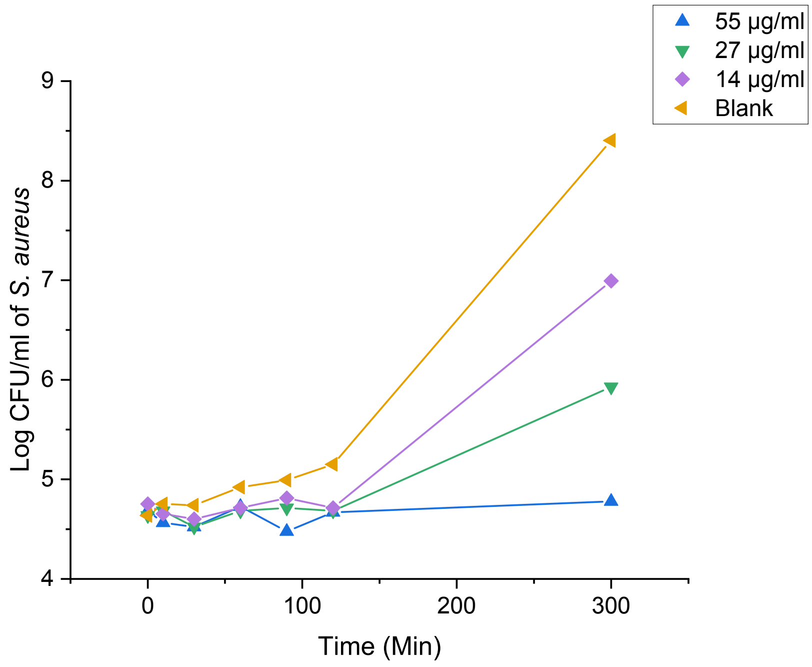

Figure S19: *S. aureus* growth curve in the presence of S50-Ger nanoparticles after 5 h incubation in nutrient-rich LB medium.

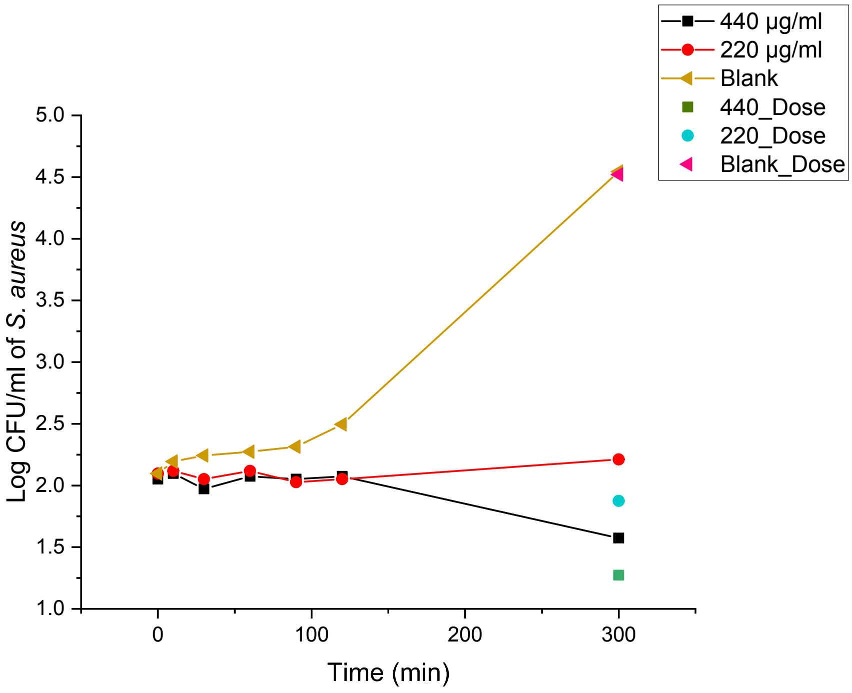

Figure S20: *S. aureus* growth curve at a lower intial cell concentration in the presence of S50-PA nanoparticles during 5 h incubation at 37 °C. Points labelled X_Dose represent time-points that were given an additional 100 µl dose of nanoparticles or blank at 120 min.

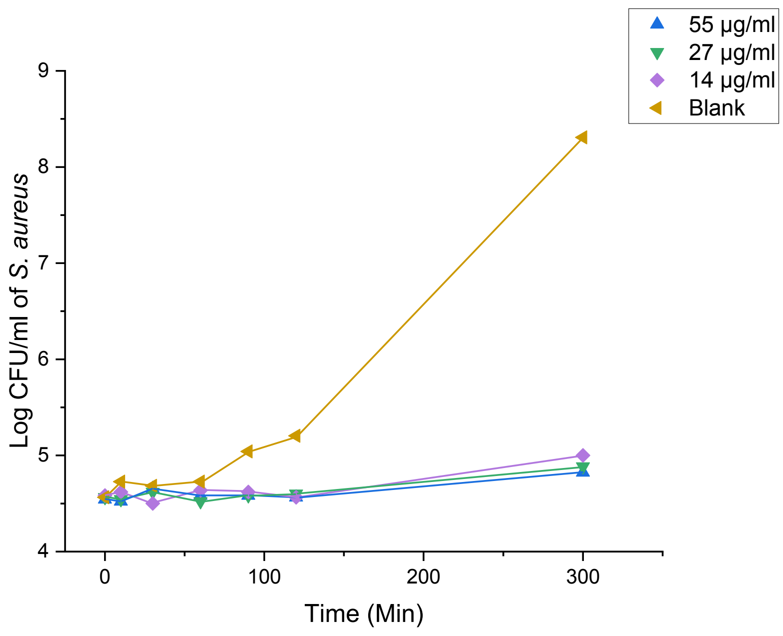

Figure S21: *S. aureus* growth curve in the presence of S70-PA nanoparticles after 5 h incubation in nutrient-rich LB medium.

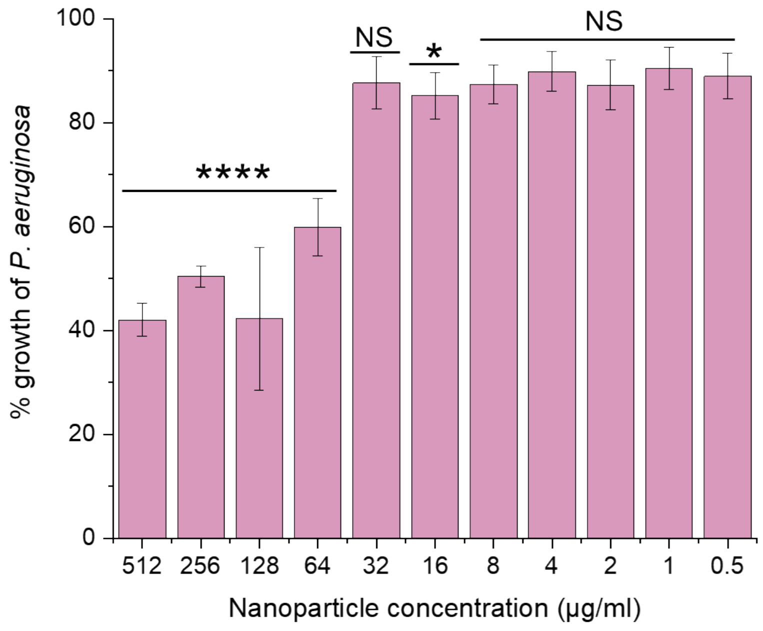

Figure S22: Graph summarising the % growth of P. aeruginosa relative to a positive control, in the presence of S50-PA nanoparticles at various concentrations during a 24 h incubation period. *$p < 0.05$, ****$p < 0.0001$, NS denotes a value that is not statistically significant compared to the control

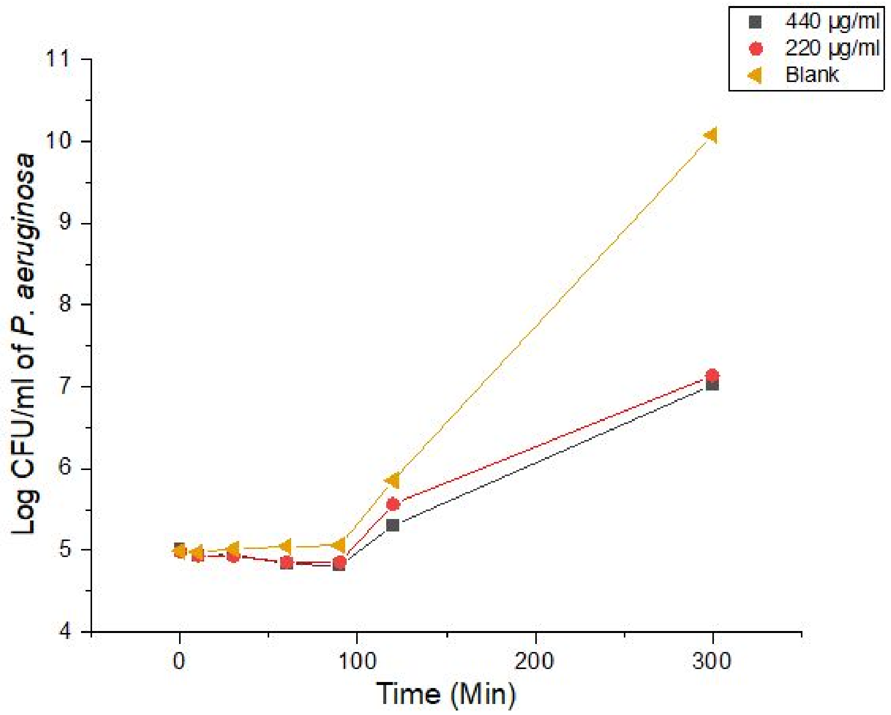

Figure S23: *P. aeruginosa* growth curve in the presence of S50-Ger nanoparticles after 5 h incubation in nutrient-rich LB medium.

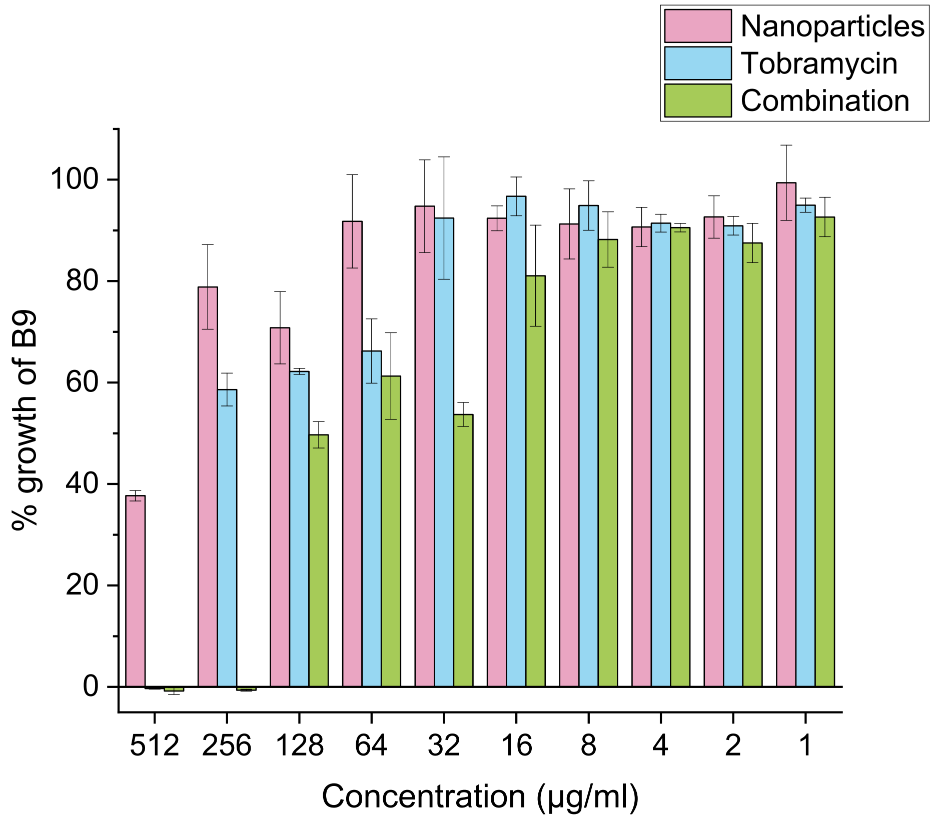

Figure S24: % growth of B9 compared to untreated culture in the presence of tobramycin (512-1 µg/ml), and a combination of tobramycin (512-1 µg/ml) and S50-PA nanoparticles (128 µg/ml).

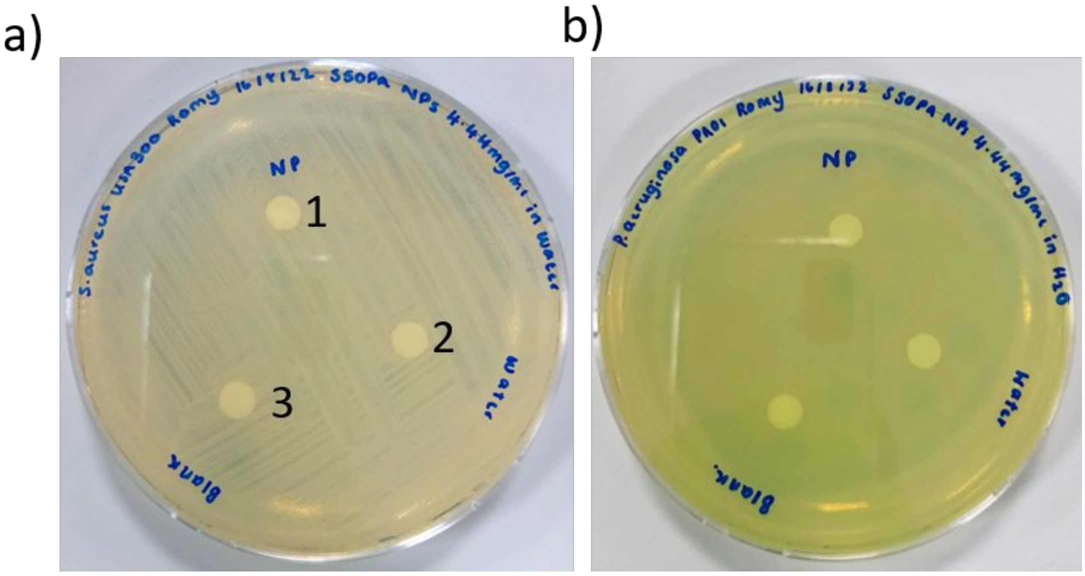

Figure S25: Image of agar plate streaked with a) *S. aureus* USA 300, and b) *P. aeruginosa* PAO1 loaded with antimicrobial susceptibility test discs containing 1) S50-PA nanoparticles 2) water and 3) empty disc.

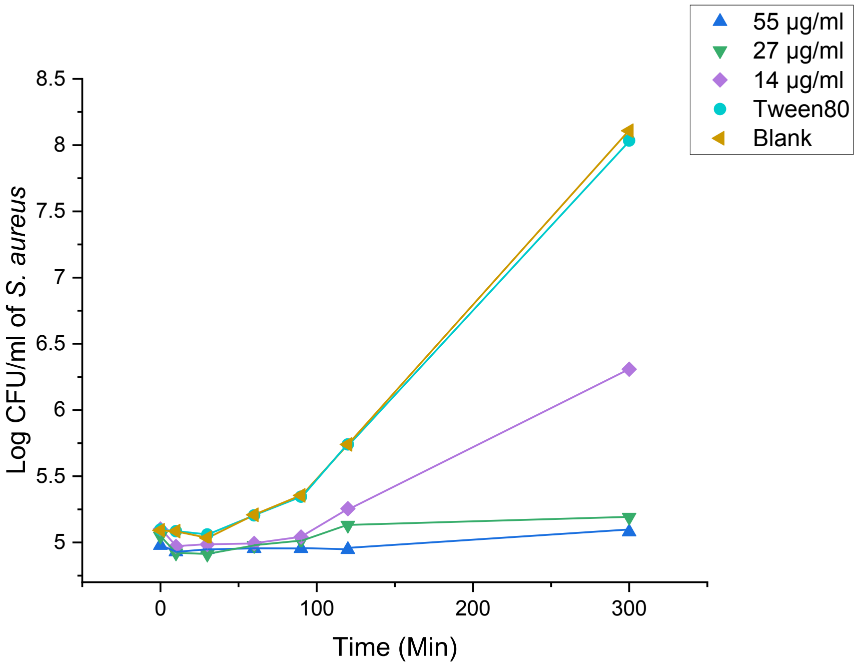

Figure S26: *S. aureus* growth curve in the presence of S50-PA nanoparticles stabilised with Tween80 over 5 h in nutrient-rich LB medium.

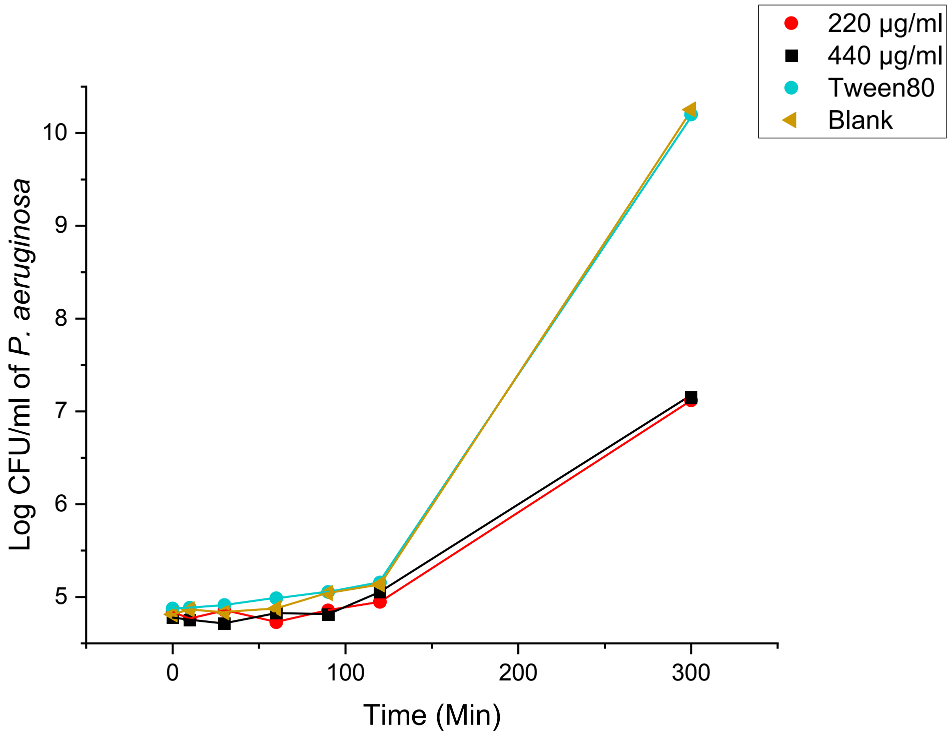

Figure S27: *P. aeruginosa* growth curve in the presence of S50-PA nanoparticles stabilised with Tween80 after 5 h incubation in nutrient-rich LB medium.

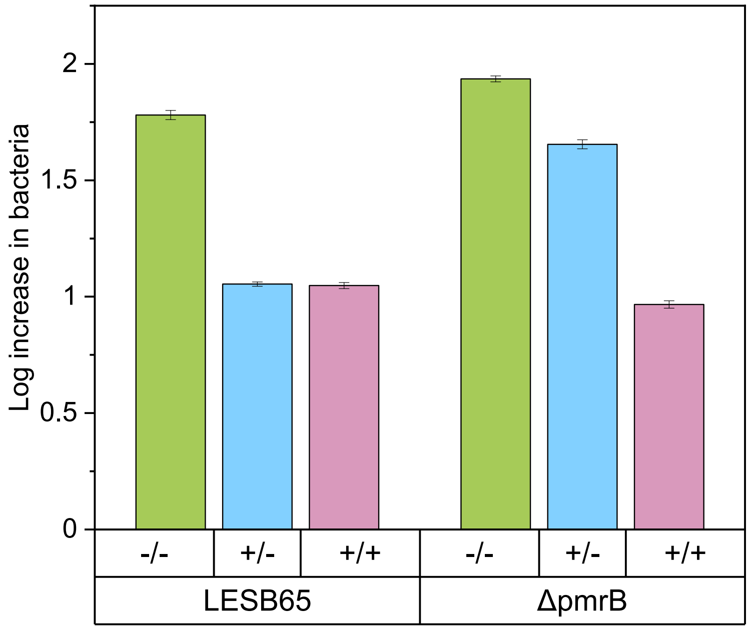

Figure S28: Log increase in LESB65 and ΔpmrB compared to initial culture for, -/- : culture only, no particles and no preincubation with spermidine, +/-: culture + nanoparticles, +/+: culture + nanoparticles + preincubation with spermidine.

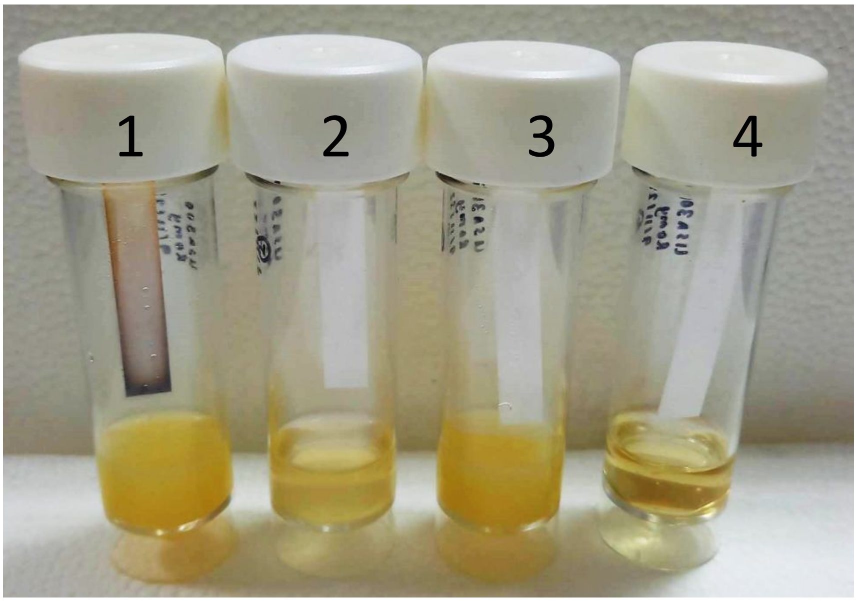

Figure S29: Lead acetate paper after 24 h exposure 1: S50-PA nanoparticles and *S. aureus* in LB medium, 2: *S. aureus* in LB medium, 3: S50-PA nanoparticles in LB medium and 4: LB medium.
